# Curcumin-based-fluorescent probes targeting ALDH1A3 as a promising tool for glioblastoma precision surgery and early diagnosis

**DOI:** 10.1038/s42003-022-03834-7

**Published:** 2022-09-01

**Authors:** Edoardo L. M. Gelardi, Diego Caprioglio, Giorgia Colombo, Erika Del Grosso, Daniele Mazzoletti, Daiana Mattoteia, Stefano Salamone, Davide M. Ferraris, Eleonora Aronica, Giulia Nato, Annalisa Buffo, Menico Rizzi, Lorenzo Magrassi, Alberto Minassi, Silvia Garavaglia

**Affiliations:** 1 Dipartimento di Scienze del Farmaco, University of Piemonte Orientale, Via Bovio, 6, 28100 Novara, Italy; 2 IXTAL srl, Via Bovio 6, 28100 Novara, Italy; 3 Department of (Neuro)Pathology, Amsterdam UMC, University of Amsterdam, Amsterdam, The Netherlands; 4 Stichting Epilepsie Instellingen Nederland (SEIN), Heemstede, The Netherlands; 5 Department of Life Sciences and System Biology, University of Turin, Via Accademia Albertina 13, Turin, Italy; 6 Neuroscience Institute Cavalieri Ottolenghi (NICO), 10043 Orbassano, TO Italy; 7 Department of Neuroscience Rita Levi Montalcini, University of Turin, Via Cherasco 15, Turin, Italy; 8 Neurosurgery, Department of Clinical, Surgical, Diagnostic and Pediatric Science, University of Pavia, Foundation IRCCS Policlinico San Matteo, 27100 Pavia, Italy; 9 Istituto Di Genetica Molecolare IGM-CNR, Via Abbiategrasso 207, 27100 Pavia, Italy; 10 PlantaChem srls, Via Canobio 4/6, 28100 Novara, Italy

**Keywords:** Isoenzymes, Surgical oncology

## Abstract

Glioblastoma (GBM) is the most aggressive primary brain tumour for which both effective treatments and efficient tools for an early-stage diagnosis are lacking. Herein, we present curcumin-based fluorescent probes that are able to bind to aldehyde dehydrogenase 1A3 (ALDH1A3), an enzyme overexpressed in glioma stem cells (GSCs) and associated with stemness and invasiveness of GBM. Two compounds are selective versus ALDH1A3, without showing any appreciable interaction with other ALDH1A isoenzymes. Indeed, their fluorescent signal is detectable only in our positive controls in vitro and absent in cells that lack ALDH1A3. Remarkably, in vivo, our Probe selectively accumulate in glioblastoma cells, allowing the identification of the growing tumour mass. The significant specificity of our compounds is the necessary premise for their further development into glioblastoma cells detecting probes to be possibly used during neurosurgical operations.

## Abstract (web summary)

Curcumin-based fluorescent probes are presented that are able to selectively bind to aldehyde dehydrogenase 1A3 (ALDH1A3), an enzyme overexpressed in glioma stem cells and specifically identify glioblastoma cells in vitro and in vivo.

## Introduction

The term Glioma refers to a class of primary brain tumors that represent the 40% of all brain tumors. Glioblastomas are characterized by highly morphological heterogeneous neoplasms. The term “Glioblastoma” is a synonym of grade IV astrocytoma (GBM multiforme, or conventional GBM accordingly to WHO classification). Two histologic variants of GBM has been recognized with different clinicopathologic properties: gliosarcoma and giant cell glioblastoma^1^. Moreover, transcriptomic profiling has permitted to divide GBMs in four significant subtypes: proneural (PN), neural, classic, and mesenchymal (Mes)^2,3^. GBMs are very invasive and associated with a high ability to metastasize, with an extremely fast cell growth, a marked chemoresistance and a poor clinical outcome^4^. Their marked drug resistance is related to the presence of a high density of cancer stem cells (CSCs) that possess self-renewal ability and an unlimited proliferative potential, just like their physiological counterparts^5,6^. Glioma stem cells (GSCs) also provide the tumor with an enhanced resistance to drugs, radiation and oxidative stress, thus increasing GBM resistance to treatments, and their presence is associated with metastasis and relapse. Moreover, mesenchymal GSCs (Mes-GSCs) proved to be significantly more radioresistant than proneural GSCs (PN-GSCs)^7–10^. It has been demonstrated that the radiation treatment can induce the phenotype shift from PN to Mes, set out by the loss of the PN marker SOX2 and by the expression of the Mes marker CD44^11^. The tumors that are rich in Mes GSCs are the deadliest, the most dangerous and the most susceptible to relapse. As far as the tumorigenic behavior is concerned, Mes-GSCs showcased a higher growth potential under identical conditions compared to PN-GSCs both in vitro and in vivo^2^. The only resolutive treatment is the surgery that, when possible, allows the complete resection of the main tumor^12,13^. Even with this approach, though, the patient lifespan is around 5 years. This is the reason why the development of innovative tools for an early diagnosis and for the chemo treatment is of the utmost importance. In a paper published by Zhang et al.^14^, a transcriptomic analysis highlights the enrichment of the cytoplasmatic enzyme aldehyde dehydrogenase 1A3 (ALDH1A3) in Mes-GSCs. ALDH1A3 belongs to an enzymatic superfamily of aldehyde dehydrogenases, composed of 19 different isoforms, and involved in the irreversible NAD^+^-dependent oxidation of a wide range of aldehydes^15^. This superfamily is also involved in the reduction of oxidative stress and in the metabolism of several drugs, such as cyclophosphamide^16^. ALDH1A3 belongs to the ALDH1A superfamily, that also includes ALDH1A1 and ALDH1A2. All three isoenzymes are involved in the oxidation of retinal to retinoic acid, a molecule that is essential for tissue differentiation and cellular development^17,18^. ALDH1A1 and ALDH1A3 have been described as important markers^19,20^ and targets^21–23^ of CSCs in a wide variety of tumors. A several number of evidence acknowledge that ALDH1A3 can be considered a characteristic hallmark of the Mes-GSCs, which may play an important role in glioma malignancy, given that it is involved in stem cell viability drug resistance and cells maintenance arguing tumor invasion^24,25^. Considering the well-known catalytic function of ALDHs, they are regarded as the key enzymes that can detoxify harmful aldehydes within the organism, and this could be the reason why so many cytotoxic antineoplastic molecules are inactivated by CSCs^26–28^. As a result, ALDH1A1 and ALDH1A3 may protect CSCs from antineoplastic molecules, their levels could represent a prognostic factor that could anticipate the chemotherapy efficacy and their inhibition could make the tumor cells susceptible to medical treatments^29–31^. To date, CSCs are considered as one of the key mechanisms used by the tumor to evade chemotherapy and radiation treatment. Based on these evidences targeting CSCs aiming to improve already-existing therapies, prevent the relapse of the tumor and facilitate an early diagnosis could improve already-existing therapies and prevent the relapse of the tumor.

Curcumin is the most famous and most abundant congener of curcuminoids, a class of bioactive compounds isolated from turmeric (*Curcuma longa* L.) and commonly used for flavoring food in the Southeast Asian and Middle Eastern countries. Nowadays it is used in the food industry as a coloring agent known as E100. In the past decade, this natural dye became one of the best candidates for the development of new therapies against gliomas^32^. The antineoplastic abilities of curcumin, such as the induction of apoptosis and the inhibition of proliferation and invasion, have been proved in several tumors, including gliomas. Curcumin is also capable of inducing reactive oxygen species (ROS) in a wide variety of cancers, so as to lead to the activation of the MAPK apoptotic pathway^33^. Even though CSCs radical scavenging systems have been reported, several studies confirmed that curcumin-induced ROS can target GSCs^34^. However, this plethora of bioactivities should be viewed with skepticism: curcumin is one of the most famous Pan-Assay Interference Compounds, exhibiting all known behaviors of this class of molecules and confining it to a mere academic curiosity^35^. Besides the well-known health-promoting benefits, curcumin also possesses a strong intrinsic fluorescence and some of its derivatives have recently been acknowledged as optical probes for the in vivo studies of several diseases, such as Alzheimer^36^ and solid tumor^37,38^.

To date, as the surgical resection of the tumor mass is the fundamental treatment for GBM, having a tool that could lead to an early diagnosis and improve the surgeon accuracy during the operation would be a great advantage^39^. Therefore, a fluorescent probe that can detect a mass of CSCs within the brain of a patient with glioma could be extremely helpful and useful. In this paper, we present the first study of two different selective ALDH1A3 fluorescent probes, with a curcumin scaffold-based nature, that are able to inhibit the activity of the recombinant enzyme and can be detectable only in our positive controls, both in vitro and in vivo.

## Results

### Chemistry

**Fig. 1:**
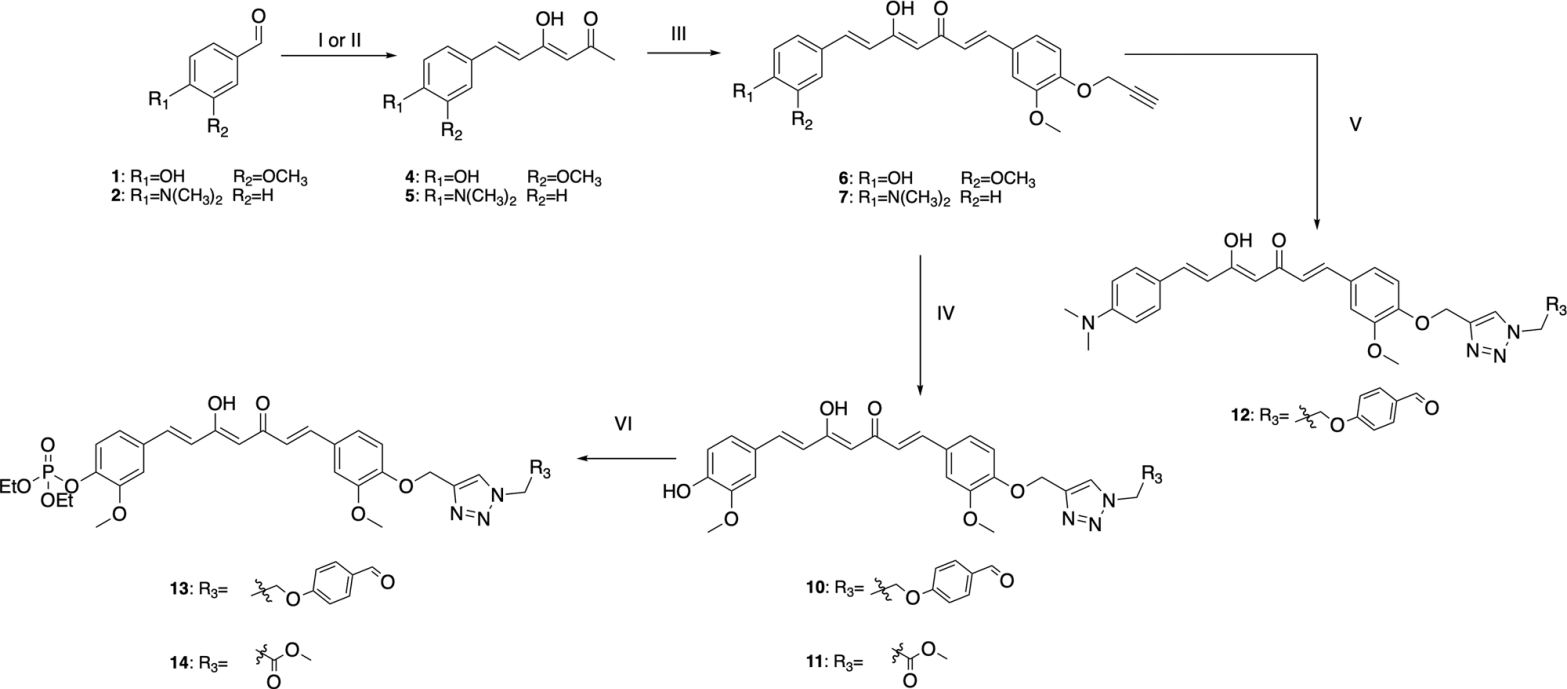
Synthesis of compounds 10, 11, 12, 13 and 14. (I) Acetylacetone, boron oxide, trimethyl borate, *n*-butylamine, DMF; (II) acetylacetone, boron oxide, trimethyl borate, *n*-butylamine, EtOAc; (III) boron oxide, trimethyl borate, *n*-butylamine, DMF; (IV) CuSO_4_, Na-ascorbate, t-BuOH/H_2_O/CH_3_CN 2:1:1; (V) CuSO_4_, Na-ascorbate, t-BuOH/H_2_O/CH_3_CN 2:1:1; (VI) diethylchlorophosphate, TEA, CH_2_Cl_2_.

In this project we propose the synthesis of a class of curcumin-based probes where a triazole moiety is used as connector between the fluorescent dye and the functional group needed for the interaction with the active site of the enzyme. Since we already worked on the curcumin scaffold^40–43^, we identified five key synthons as starting points: the hemi-curcuminoids **4** and **5**, the *O-*propargyl-vanillin (**3**) and the azido derivatives **8** and **9**. Compounds **4** and **5** were easily obtained condensing respectively vanillin (**1**) and 4-(dimethylamino)benzaldehyde (**2**) with acetylacetone under Pabon conditions^44^. The replacement of the vanillic moiety with a dimetylamino group was done to modulate the fluorescence emission^45^ and, in the attempt to enhance the solubility of the final compound. A second Pabon condensation of hemi-curcuminoids **4** and **5** with *O*-propargyl-vanillin (**3**), furnished respectively compounds **6** and **7** that underwent to a copper catalyzed Huisgen [3 + 2] cycloaddition with **8** and **9** leading compounds **10**, **11** and **12**. Given the possible solubility problems due to the presence of a triazole moiety together with a curcuminoid sub-structure^46,47^, compounds **10** and **11** were phosphorylated using diethylchlorophosphate in presence of triethylamine affording compounds **13** and **14** (Fig. 1 and Supplementary Fig. S1).

### Biochemical characterization of the interaction of probe 10 and probe 11 with recombinant human ALDH1A3

**Fig. 2:**
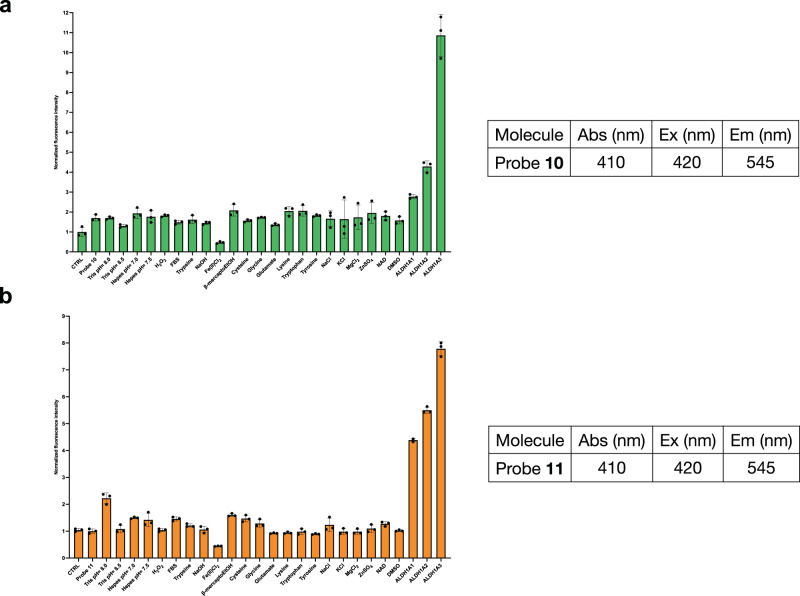
Biochemical characterization of the interaction of probe **10** with human ALDH1A3. **a** Increase of Probe **10** fluorescence intensity in presence of various biomolecules reactive at concentrations of 100 μM. For all assays, Probe **10** was used at 10 μM final concentration and valuation of the absorbance, excitation and emission wavelengths of the compound Probe **10** at a fixed concentration of 10 µM in the SEC buffer. **b** Increase of Probe **11** fluorescence intensity in presence of various biomolecules reactive at concentrations of 100 μM and evaluation of the absorbance, excitation, and emission wavelengths of the compound Probe **11** at a fixed concentration of 10 µM in the SEC buffer.

All five fluorescent compounds were firstly characterized for their solubility in physiological buffer and for their absorbance values. We then performed a 3D analysis using a TECAN SPARK to simultaneously evaluate the excitation and the emission wavelengths, that were supposed to be similar to the curcumin ones^48^. Among all five molecules, the Probe **10** and **11** showed the highest solubility and their fluorescence values were similar to the not substituted fluorophore. For these reasons, we decided to continue the biochemical characterization on Probe **10** and **11** using the selected parameters (Fig. 2). The fluorescent signal changes for both molecules upon binding to the target, were firstly evaluated with recombinant human ALDH1A3. Prior to the interaction with the target protein, the two probes possess an intrinsic low fluorescence emission, but the subsequent addition of ALDH1A3 to the mix leads to a consistent increase in the intensity of the fluorescent signal of about 12 folds for Probe **10** and 8 folds for Probe **11** (Fig. 2). To better characterized our fluorescent compounds, we tested their potential cross-reactivity toward the other two isoforms of the ALDH1A subfamily. As shown in Fig. 2, Probe **10** exhibits low cross-reactivity with the other two isoforms, with a reduction of the fluorescence signal of about 2 folds for ALDH1A1 and 4 folds for ALDH1A2, compared to what observed with ALDH1A3. In the case of probe **11**, 4 folds and 6 folds reduction of fluorescence was observed in the presence of ALDH1A1 and ALDH1A2, respectively, compared to what observed with ALDH1A3. Both probes therefore revealed selectivity for ALDH1A3. In addition, a wide range of biologically relevant analytes were tested at a concentration of 100 μM in complex with a fixed concentration of 10 μM of the probe (either probe **10** or probe **11**), to make sure that no potential off-target signals could affect the analysis. More specifically, we checked if there were abnormal fluorescence signals that could have been contributed by the presence of biologically relevant compounds, (e.g., biologically relevant amino acids), to buffers and molecules commonly used in experimental procedures. None of the selected compounds generated significant fluorescent signals, compared with the positive controls (Fig. 2).

**Fig. 3:**
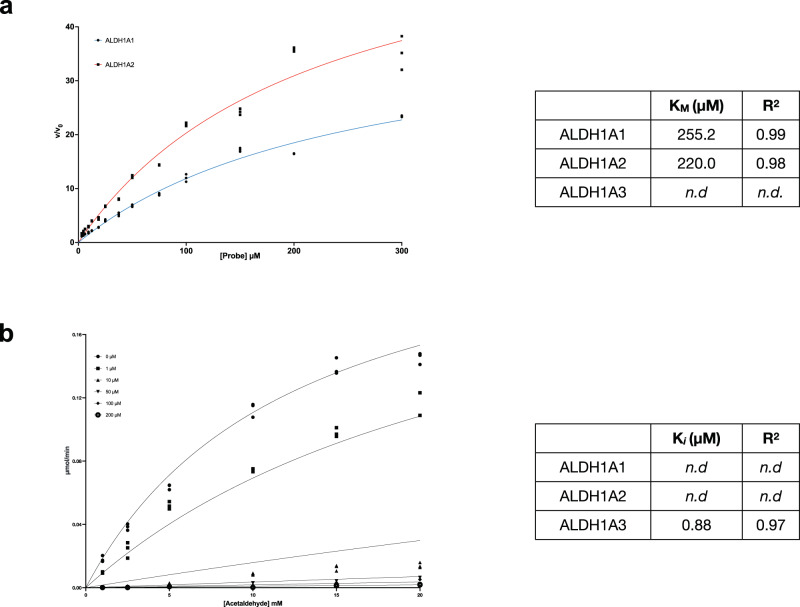
Kinetic characterization of the interaction of probe **10** with human ALDH1A3. **a** *K*_M_ graph and value of the complex between Probe **10** at fixed concentration and various concentration of ALDH1A1 and ALDH1A2. **b** *K*_i_ graph and value of the complex between Probe **10** and ALDH1A3, in presence of different substrate and probe concentration. Probe **10** was analyzed as potential full competitive inhibitor.

**Fig. 4:**
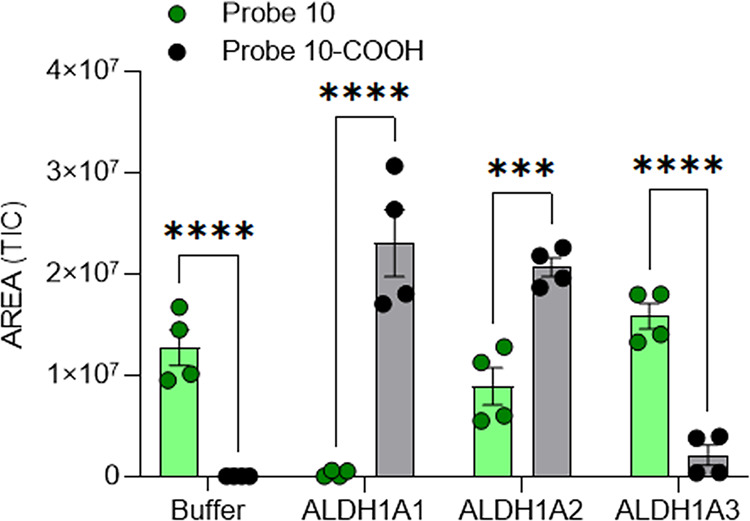
LC-HRMS analysis for the determination of interaction of Probe **10** with the different isoenzymes of ALDH1A. LC-HRMS results after 1 h of incubation of single isoenzyme with Probe **10** in presence of ALDH1A1, ALDH1A2 and ALDH1A3. It is possible to appreciate the formation of the corresponding acid (Probe **10-COOH**) for 1A1 and 1A2 but not for ALDH1A3. (TIC total ion current).

On the basis of these encouraging results, we conducted an in-depth biochemical analysis on the two probes in order to characterize their mechanism of action. For greater clarity we will first illustrate the results obtained for Probe **10** and then those for Probe **11**. The affinity of Probe **10** to the different isoenzymes revealed highly similar *K*_d_ values for all the three forms (ALDH1A1 = 31.1 μM *R*^2^ = 0.98, ALDH1A2 = 36.9 μM *R*^2^ = 0.98 and ALDH1A3 = 38.2 μM *R*^2^ = 0.96). To clearly understand the nature of the interaction between Probe **10** and ALDH1A3 we tested our fluorescent compound as an inhibitor, using an already published protocol^17^. As shown in Fig. 3a, ALDH1A3 resulted to be the only strongly inhibited isozyme, with a *K*_i_ value of 0.880 μM (*R*^2^ = 0.97) and a competitive mechanism of action. Neither of the two other isozymes were inhibited, even at the highest probe concentration used to test the catalytic activities of ALDH1A1 and ALDH1A2. Probe **10** was also tested as a possible substrate, due to the presence of a benzaldehyde on the lateral chain of the fluorophore (Fig. 3b). Indeed, after 1 h of incubation, Probe **10** was fully metabolized and converted to its carboxylic derivative (Probe **10-COOH**) by both ALDH1A1 and in part by ALDH1A2, while no oxidation could be detected in the case of ALDH1A3 as unambiguously demonstrated by a LC-HRMS analysis (Fig. 4 and Supplementary Fig. S2.a). Our investigations clearly demonstrate that Probe **10** binds to the enzyme active site without undergoing oxidation. All the results obtained allow us to understand and explain why it shows the same affinity toward the three isoenzymes: it is a substrate for ALDH1A1 and ALDH1A2 and a potent competitive inhibitor for the isoform 1A3. In conclusion, the isoform 1A3 is inhibited by Probe **10** in the low micromolar range, shows selectivity vs. the other two isoforms and its binding to the enzyme active site results in a significant increase of the fluorescence signal.

As described above for molecule **10**, the affinity of Probe **11** for the three isozymes has also been investigated by determining the *K*_d_ of the compound-enzyme complexes. Probe **11** preferentially interacts with ALDH1A1 and ALDH1A3, with similar *K*_d_ values, while is less affine to the ALDH1A2 isoform (Supplementary Fig. S3). Surprisingly, Probe **11** has no inhibitory effect against any of the three ALDH1A isoenzymes and has not been shown to be a substrate. These results have been further confirmed by LC-HRMS experiments carried out on Probe **11** in the presence of the three different isoenzymes (Supplementary Fig. S2.b). Overall, our data suggest that Probe **11** does not display a strong selectivity among the three enzymes but rather behaves as a pan-probe for the ALDH1A subfamily.

Taken together, and from a biochemical perspective, these results suggest that both compounds are suitable for our aims with Probe **10** that emerges as the best performer. Yet, a much deeper in vitro analysis is needed to confirm the potential selectivity of Probe **10** and Probe **11** and to better investigate the possible cytotoxic effects.

### ALDH1A3 detection in vitro

**Fig. 5:**
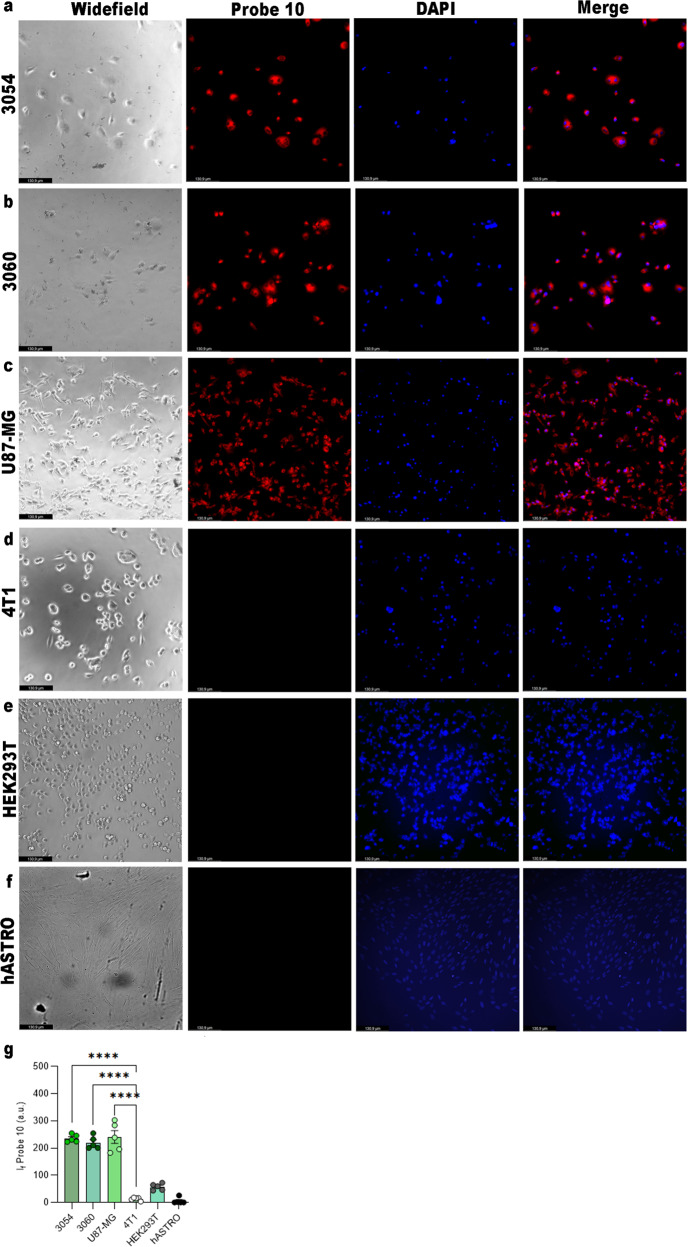
Probe** 10** is selective on glioblastoma ALDH1A3 positive cells. Fluorescence microscope images for Probe **10** detection in **a** patient-derived glioma cell 3054, **b** 3060, **c** U87MG, **d** 4T1, **e** HEK293T and **f** normal hASTRO at 10 µM, after 2 h of incubation. **g** Relative fluorescence intensity of the images acquired. ****p value <0.001.

**Fig. 6:**
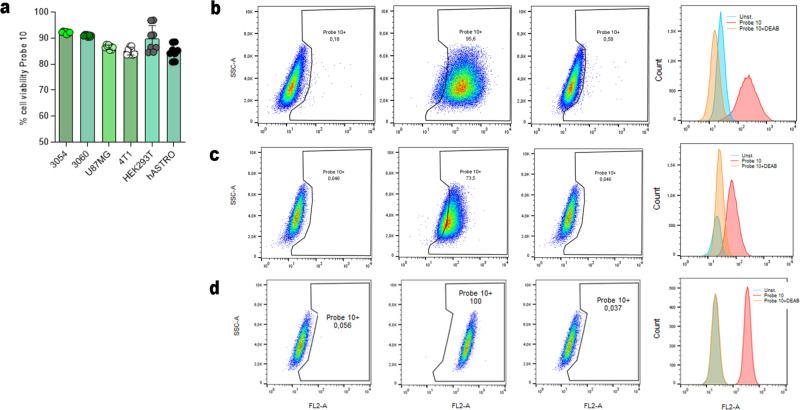
Application of Probe **10** in glioblastoma cell lines. **a** Cell viability at 10 µM of Probe **10** at 72 h. Flow cytometry analysis of **b** 3054, **c** 3060, **d** U87-MG cells unstained, stained with Probe **11** (10 µM) with or without DEAB (1 µM). On the right of each panel of flow cytometry histographic profiles of unstained, stained with Probe 10 (10 µM) with or without DEAB (1 µM) is reported.

To better characterized the selectivity fluorescence profile of Probe **10**, we selected four cell lines based on their different ALDH1As expression profile, as described in The Human Protein Atlas (https://www.proteinatlas.org/), in order to validate the in vitro behavior of the two probes. Human U87MG glioblastoma cells are labeled as ALDH1A3^+^ cell line, HEK293T as ALDH1A2^+^ cell line, human fetal astrocytes (hASTRO) as ALDH1A1^+^ cell line and 4T1 mammary carcinoma as triple negative ALDH1As subfamily^23^. Moreover, we have chosen two different patient-derived glioblastoma cell lines (3054 and 3060) according to their increased expression levels of ALDH1A3 compared to the other ALDH1A isoforms (ALDH1A3 fold change of 8.58 and 7.34, ALDH1A2 fold change 5.8 and 5.6, ALDH1A1 4.1 and 4.5, respectively, as reported in HGCC site https://www.hgcc.se/). We determined the ability of Probe **10** to enter the cells and verified its fluorescence in ALDH1A3 positive cells. As depicted in Fig. 5a, b, Probe **10** can determine fluorescence in patient-derived glioblastoma cell lines 3054, 3060 and in the immortalized line U87MG, but not in HEK293T, hASTRO and 4T1 cell lines (Fig. 5c–e). The probe is localized to the cytoplasm confirming the specific and exclusively cytosolic binding to ALDH1A3, as confirmed with fluorescence quantification (Fig. 5g). At the same time, Probe **10** is unable to induce cell mortality (Fig. 6a). These data were corroborated by the results of flow cytometry analysis (Fig. 6b–d and Supplementary Fig. S4), showing that positivity to Probe **10** was limited to ALDH1A3^+^ cells. Positivity reverted with pre-treatment by the pan-ALDH inhibitor DEAB. Moreover, the fluorescence has been observed using Probe **11** only in U87MG cell lines (Supplementary Fig. S5a–d).

### Probe 10 is able to selectively define glioblastoma cells in vivo

**Fig. 7:**
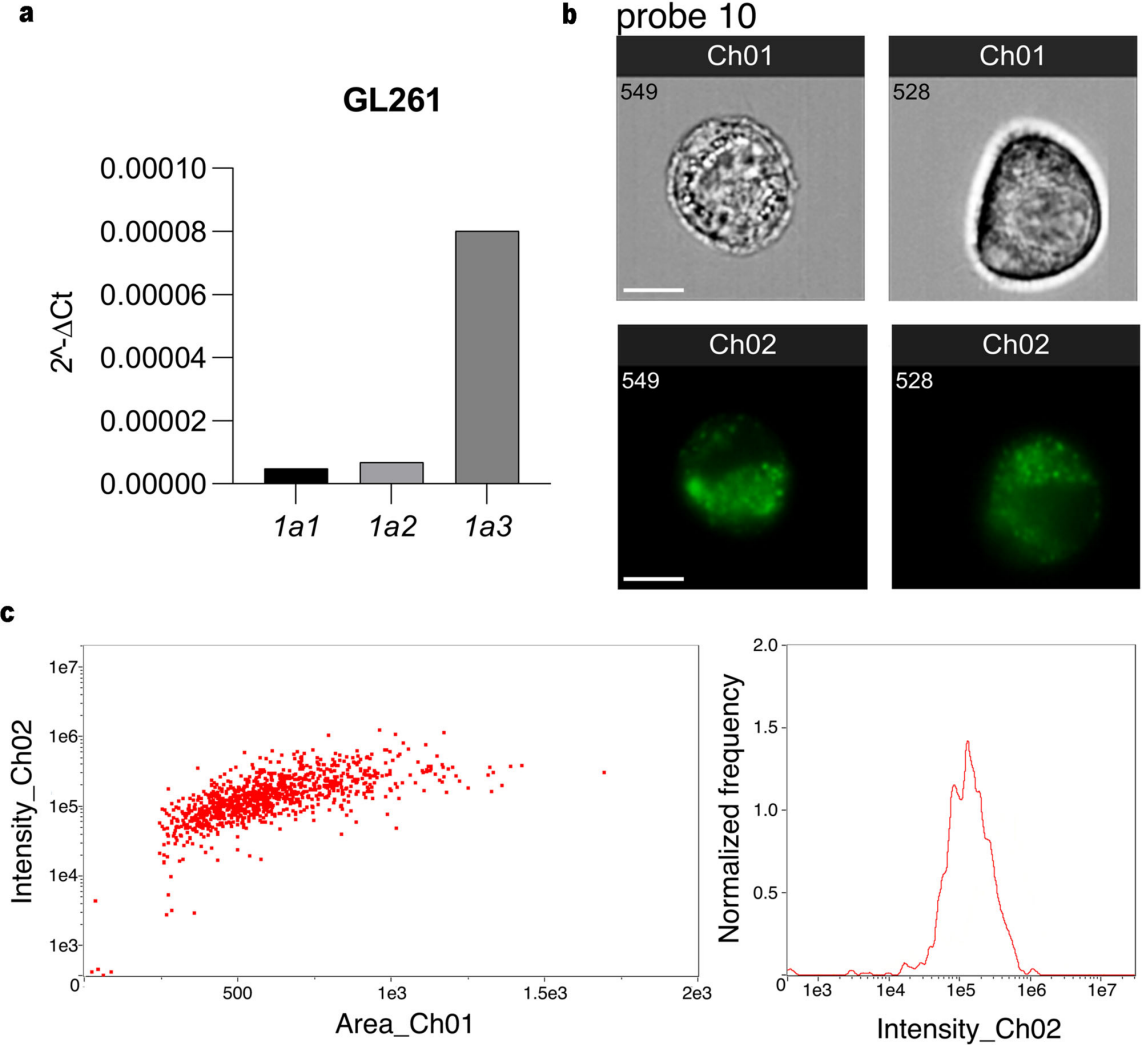
In vitro experiments showing that ALDH1A3 is the most expressed isoform in GL261 murine glioma cells and both fluorescent substrates enter and label the same cells. **a** Results of quantitative rtPCR showing the levels of expression of ALDH1A1 (1a1), ALDH1A2 (1a2) and ALDH1A3 (1a3) in murine GL261 cells. ALDH1A3 is significantly more expressed than the other isoforms. **b** Living GL261 cells were analyzed through an ImageStreamX MarkII using two channels: brightfield (Ch 01) and fluorescence (Ch 02) after 1 h incubation in probe 10, cells were thoroughly washed in PBS and analyzed. Two different representative cells are shown. From above, row 1 brightfield images (Ch 01), row 2 fluorescent images (Ch 02). “In Focus Cells” were identified based on the “Gradient Root Mean Square (RMS) Contrast Feature” that captures in focus images of cells identified by high normalized pixel intensity gradient (RMS values) derived from Ch 01; then, a scatter plot of the “Aspect Ratio Feature” vs. brightfield “Area Feature” was used to identify single cells (singlets) from debris or cell clumps based on high aspect ratio and low area value. **c** Fluorescence intensity variations are shown in the histogram and dot plot (Area vs. Intensity). Scale bars: 5 µm are the same in all images.

**Fig. 8:**
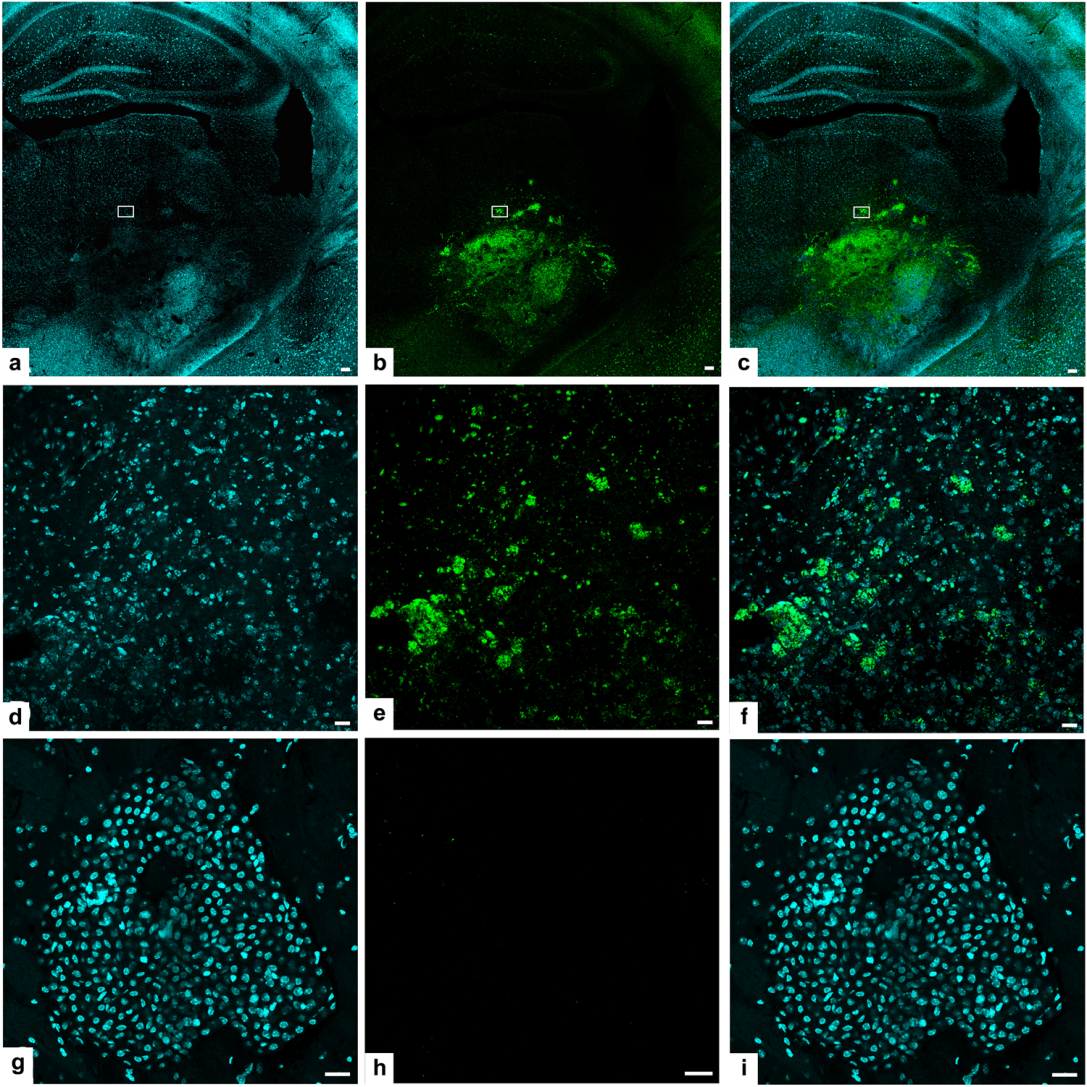
In vivo labeling by the fluorescent ALDH1A3 substrates of GL261 cells growing and infiltrating the host brain 6 days after the initial inoculum. **a**–**f** Confocal images showing the same coronal section of a tumor-bearing brain in an animal. injected i.p. with Probe **10** for 6 h. In all images the area boxed is the same and it is shown at higher magnification in **d**–**f**. In **a** cell nuclei are stained by DAPI, in 6b, GL261 cells containing Probe **10** fluorescence in green. Most of the fluorescence comes from glioma cells growing in the left striatum and adjacent structures that have taken up and metabolized Probe **10**. Scale bars: 25 μM. In 6c, the image shows the double fluorescence of DAPI and Probe **10** together. **d**–**f** show confocal images showing at higher magnification the area boxed in 6a–c. Fluorescence of Probe **10** is mostly contained in the cytoplasm. Scale bars: 25 μM. **g**–**h** Confocal images showing, under different fluorescence conditions, the same coronal section of a tumor-bearing brain in an animal i.p. injected with compound Probe **11** that does not penetrate to any appreciable level the tumor cells in vivo, despite penetrating GL261 cells in vitro. Scale bars: 25 μM. **i** Confocal image under excitation and filtering conditions appropriate both for DAPI and Probe **11**, no fluorescence due to Probe **11** is visible despite the abundance of tumor cells.

Based on the excellent results obtained from the in vitro analyses, we decided to verify the potential of our probes also in vivo. The presence of a mouse ortholog of our target enzyme, ALDH1A3, having a sequence homology equal to 98%, allowed us to set up an in vivo experiment. To evaluate the fluorescent signal of both probes in an in vivo model of orthotopic transplantation of glioblastoma cells, we decided to use the GL261, a murine transplantable high grade glioma cell line positive to ALDH1A3. We initially measured the mRNA of the enzyme of our interest and checked whether the GL261 cells show the same permeability to our fluorescent compounds (Fig. 7a and Supplementary Fig. S6). Figure 7b, c and Supplementary Fig. S6 show that also in GL261 cells both Probes **10** and **11** were able to enter and label the murine glioblastoma cells. In particular, Probe **11** in vitro displays a higher intrinsic fluorescence, but it is less selective due to the presence of background signals that are non-specific for cancer cells internalization, as already suggested by the biochemical experiments. Nevertheless, even if the fluorescent signal has turned out to be lower in vitro, Probe **10** appears to be more promising, due to its ability to accumulate in tumor cells with a lower background signal. Subsequently, we induced glioblastoma in mice brain by stereotactically injecting 1 × 10^5^ GL261 cells into the left striatum of adult mice. All mice were injected i.p. with an equivalent dose of the 2 probes. The in vivo experiments showed that only Probe **10** was able to selectively label the growing tumor. As shown in Fig. 8a–c, Probe **10** accumulates in GL261 cells outlining the tumor growing in the left striatum and invading the adjacent areas of the brain, without significant interference from the adjacent tissue. Scattered tumor cells were also visualized infiltrating the adjacent areas of the mouse brain. In addition, it is possible to appreciate that in tumor the fluorescence of Probe **10** is mostly contained in the cytoplasm of cancer cells (Fig. 8d–f). The same coronal section of a tumor-bearing brain in an animal injected with compound Probe **11** does not penetrate to any appreciable level the tumor cells in vivo, despite penetrating GL261 cells in vitro (Fig. 8g–i).

## Discussion

The research of new targets for anti-cancer therapies and of markers for an efficient surgery and diagnosis still represents an ongoing challenge for several tumors and in particular for glioblastoma. With this paper, we describe new possible selective probes that are able to detect ALDH1A3 in GBM cancer cells. Both fluorescent compounds showed biological interesting data, highlighting Probe **10** as a promising starting point the development of ALDH1As probes. Indeed, this molecule can be considered as the first selective fluorescent probe for human ALDH1A3 ever reported and characterized in a specific cancer cell line. The biochemical characterization of Probe **10** demonstrates its selectivity toward ALDH1A3, and our in vitro experiments confirmed a strong signal not only in U87 cell line, ALDH1A3^+^, but also in the patient-derived glioblastoma cell lines 3054 and 3060, compared with our negative controls where no significant emission was observed, meaning while, the in vitro imaging using the 4T1 cell line, ALDH1A^−^, but ALDHs positive, shows only unspecific signals. Moreover, fluorescence emission in ALDH1A3^+^ glioblastoma cell lines is only present in the cytosol, while the nucleus appeared unstained. Probe **10** is characterized by a benzaldehyde moiety on the lateral chain that is also present in DEAB^49^. Based on this chemical feature, Probe **10** showed a marked binding preference for ALDH1As, with highly similar *K*_D_ values. Our biochemical data confirmed that Probe **10** binds to the catalytic site of all three isoenzyme and it is metabolized in its corresponding acidic form by ALDH1A1 and ALDH1A2 but not by ALDH1A3. In the latter, Probe **10** was observed to behave as a strong competitive inhibitor with *K*_i_ value in the low micromolar range (0.880 μM). As confirmed by the in vitro imaging analysis, Probe **10** was detectable only in our positive controls U87MG, 3054 and 3060, whereas in the negative controls we were not able to detect any significant cytosolic emission. As a result, the Probe **10** displays the characteristics of an inhibitor of ALDH1A3 that most probably directly interact with the catalytic cysteine, Cys 314, while the other two isoenzymes metabolize the molecule in a faster way. We also performed a flow cytometry analysis that showed that Probe **10** is more selective than Probe **11** on the U87 ALDH1A3^+^ cells, even with a lower fluorescent intensity. As show in result section, we confirmed our data also in vivo on model of orthotopic transplantation of GL261 glioblastoma cells, positive at the ortholog of human ALDH1A3. Our results clearly show that Probe **10** is able to delimit the cancerous mass. Indeed, we obtained a significant signal only delimited on the tumor tissue in treated animals. This property could be exploited to improve precision in fluorescence-guided resection of malignant gliomas. This is a rapidly evolving technique that is currently based on the injection of fluorescent markers like fluorescein and 5-aminolevulinic acid that accumulate in the tumor by unspecific mechanisms^50–52^. The finding of new markers, with the aim of designing more specific molecules for more effective therapies and faster diagnosis, is essential for the modern precision medicine and ALDH1A3 represents one of the most suitable candidates for this goal. Taken together, our results highlight that Probe **10** is a promising tool to selectively sort glioblastoma infiltrating cells, if compared with the probes already available on the market, such as ALDEFLOUR.

In conclusion, our results demonstrate that Probe **10** is the first ALDH1A3 selective fluorescent tool that is able to preferentially bind to the target enzyme, without inducing significant cytotoxic effects, both in vitro, in human Glioblastoma cells U87MG, 3054, 3060 and in vitro and in vivo murine GL261 cells outlining the tumor growing in the left striatum and invading the adjacent areas of the brain.

## Materials and methods

### General

NMR spectra were measured on Bruker Avance 400 MHz spectrometer. Chemical shifts were referenced to the residual solvent signal (CDCl_3_: *δ*_H_ = 7.21, *δ*_C_ = 77.0). MS spectra were acquired by Thermo Scientific Q-Exactive Plus (Supporting Information S1). Reactions were monitored by thin-layer chromatography on Merck 60 F254 (0.25 mm) plates, visualized by staining with 5% H_2_SO_4_ in EtOH and heating. Organic phases were dried with Na_2_SO_4_ before evaporation. Chemical reagents and solvents were purchased from Sigma-Aldrich, TCI Europe or Fluorochem and were used without further purification unless stated otherwise. Petroleum ether with boiling point of 40–60 °C was used. Silica gel 60 (70–230 mesh) was used for gravity column chromatography.

### 3-methoxy-4-propargyloxybenzaldehyde (3)

Propargyl bromide (6.01 ml, 80% w/v in toluene, 52.580 mmol, 2 eq) was added to a suspension of vanillin (**1**, 4 g, 26.290 mmol, 1 eq) and potassium carbonate (5.09 g, 36.806 mmol, 1.4 eq) in acetone (80 ml). The suspension was heated to reflux for 12 h and the solvent was removed under reduced pressure. Water was added and the aqueous phase was extracted with EtOAc, washed with water, brine and dried. The crude was purified by column chromatography (PE/EtOAc 9:1 as eluent) to give 3-methoxy-4-propargyloxybenzaldehyde (**3**, 2.58 g, 52%) as white crystalline solid. ^1^H NMR (400 MHz, CDCl_3_) *δ* (400 MHz, CDCI_3_) 9.87 (s, 1H), 7.47 (dd, *J*_1_ = 6.8, *J*_2_ = 1.4 Hz, 1H), 7.44 (d, *J* = 1.4 Hz, 1H), 7.14 (d, *J* = 6.8 Hz, 1H), 4.86 (d, *J* = 2.5 Hz, 2H), 3.95 (s, 3H), 2.56 (t, *J* = 2.5 Hz, 1H). ^13^C NMR (100 MHz, CDCl_3_) *δ* 190.9, 152.1, 150.0, 130.9, 126.3, 112.5, 109.4, 77.4, 77.2, 56.6, 56.0; HRESIMS *m*/*z* [M + H]^+^ 191.0628 (calcd for C_11_H_11_O_3_, 191.0630).

### (*E*)-6-(4-hydroxy-3-methoxyphenyl)hex-5-ene-2,4-dione (4)

Boron oxide (14.6 g, 174.85 mmol, 5.3 eq) and acetylacetone (13.5 ml, 131.46 mmol, 4 eq) were dissolved in DMF (10 ml) stirred at 80 °C for 1 h. The mixture was cooled to 0 °C, then trimethyl borate (23.8 ml, 209.55 mmol, 6.38 eq) and vanillin (**1**, 5 g, 32.86 mmol, 1 eq) were added. Butylamine (1.3 ml, 13.27 mmol, 0.4 eq) was then added dropwise. The obtained mixture was heated to 80 °C for 24 h, then cooled to room temperature and 5% AcOH_aq_ (200 ml) was added. The suspension was then stirred for 2 h at room temperature and a yellow precipitate formed. The precipitate was filtered, washed with water (3 × 100 ml), and purified by chromatography over silica (PE/EtOAc 7:3) to afford (*E*)-6-(4-hydroxy-3-methoxyphenyl)hex-5-ene-2,4-dione (**4**, 3.22 g, 51%) as a yellow solid. ^1^H NMR (400 MHz, CDCl_3_) *δ* (400 MHz, CDCI_3_) 7.53 (1H, d, *J* = 15.8 Hz, 1H), 7.09 (1H, dd, *J* = 8.2 Hz, *J* = 1.9 Hz, 1H), 7.02 (d, *J* = 1.9 Hz, 1H), 6.92 (d, *J* = 8.2 Hz, 1H), 6.32 (d, *J* = 15.8 Hz, 1H), 5.91 (1H, bs, OH), 5.62 (s, 1H), 3.94 (s, 3H), 2.16 (s, 3H). ^13^C NMR (100 MHz, CDCl_3_) *δ* 197.0, 177.9, 147.7, 146.7, 140.0, 127.6, 122.6, 120.2, 114.8, 109.5, 100.7, 55.9, 26.5; HRESIMS *m*/*z* [M + H]^+^ 235.0973 (calcd for C_13_H_15_O_4_, 235.0970).

### 1-(4-(Dimethylamino)phenyl)-5-hydroxyhexa-1,4-dien-3-one (5)

Boron oxide (4.17 g, 46.933 mmol, 0.4 eq) and 4-dimethylaminobenzaldehyde (**2**, 1 g, 6.705 mmol, 1 eq) were dissolved in EtOAc (50 ml) stirred at 80 °C for 1 h. The mixture was cooled to 0 °C, then trimethyl borate (761 ml, 6.705 mmol, 1 eq) and acetylacetone (6.88 ml, 67.047 mmol, 10 eq) were added. Butylamine (665 ml, 6.705 mmol, 1 eq) was then added dropwise. The obtained mixture was heated to 80 °C for 72 h, then cooled to room temperature. H_2_SO_4_ 2 M (50 ml) was added, then the organic phase was washed with NaHCO_3_ s.s. (50 ml), water, brine and dried. The crude was purified by column chromatography (PE/EtOAc 9:1 as eluent) to afford 1-(4-(Dimethylamino)phenyl)-5-hydroxyhexa-1,4-dien-3-one (**5**, 291 mg, 19% yield) as a red powder. ^1^H NMR (400 MHz, CDCl_3_) *δ* 7.38 (s, 1H), 7.30 (d, *J* = 9.3 Hz, 2H), 6.64 (d, *J* = 9.3 Hz, 2H), 3.04 (s, 6H), 1.90 (d, *J* = 5.7 Hz, 6H); ^13^C NMR (100 MHz, CDCl_3_) *δ* 195.59, 179.56, 151.65, 140.98, 129.79, 122.79, 117.37, 111.92, 100.16, 40.11, 26.47; HRESIMS *m*/*z* [M + H]^+^ 232.1336 (calcd for C_14_H_18_NO_2_, 232.1338).

### (1*E*,4*Z*,6*E*)-5-hydroxy-1-(4-hydroxy-3-methoxyphenyl)-7-(3-methoxy-4-(prop-2-yn-1-yloxy)phenyl)hepta-1,4,6-trien-3-one (6)

Boron oxide (142 mg, 1.708 mmol, 0.4 eq) and **4** (1 g, 4.269 mmol, 1 eq) were dissolved in DMF (2 ml) stirred at 80 °C for 1 h. The mixture was cooled to 0 °C, then trimethyl borate (810 ml, 7.129 mmol, 1.67 eq) and **3** (893 mg, 4.696 mmol, 1.1 eq) were added. Butylamine (7 ml, 0.071 mmol, 0.17 eq) was then added dropwise. The obtained mixture was heated to 80 °C for 24 h, then cooled to room temperature and 5% AcOH_aq_ (50 ml) was added. The suspension was then stirred for 2 h at room temperature and an orange precipitate formed. The precipitate was filtered, washed with water (3 × 100 ml), and purified by chromatography over silica (PE/EtOAc 6:4) to afford (1*E*,4*Z*,6*E*)-5-hydroxy-1-(4-hydroxy-3-methoxyphenyl)-7-(3-methoxy-4-(prop-2-yn-1-yloxy)phenyl)hepta-1,4,6-trien-3-one (**6**, 1.21 g, 72%) as an orange solid. ^1^H NMR (400 MHz, CDCl_3_) *δ* 7.59 (d, *J* = 15.7 Hz, 2H), 7.08–7.12 (m, 3H), 7.04 (s, 2H), 6.92 (d, *J* = 8.2 Hz, 1H), 6.45–6.51 (m, 2H), 5.81 (s, 1H), 4.79 (d, *J* = 2.3 Hz, 2H), 3.92 (d, *J* = 6.2 Hz, 6H), 2.53 (t, *J* = 4.84, 3H); ^13^C NMR (100 MHz, CDCl_3_) *δ* 183.72, 182.82, 149.75, 148.65, 148.01, 146.90, 140.81, 140.11, 129.23, 127.59, 122.95, 122.54, 122.06, 121.73, 114.93, 113.77, 110.44, 109.76, 101.39, 78.09, 76.30, 56.63, 55.99, 55.97; HRESIMS *m*/*z* [M + H]^+^ 407.1496 (calcd for C_24_H_23_O_6_, 407.1495).

### (1*E*,4*Z*,6*E*)-1-(4-(dimethylamino)phenyl)-5-hydroxy-7-(3-methoxy-4-(prop-2-yn-1-yloxy)phenyl)hepta-1,4,6-trien-3-one (7)

Boron oxide (17 mg, 0.223 mmol, 0.4 eq) and **5** (130 mg, 0.558 mmol, 1 eq) were dissolved in EtOAc (3 ml) stirred at 80 °C for 1 h. The mixture was cooled to 0 °C, then trimethyl borate (191 ml, 0.949 mmol, 1.67 eq) and **3** (106, 0.558 mmol, 1 eq) were added. Butylamine (9 ml, 0.095 mmol, 0.17 eq) was then added dropwise. The obtained mixture was heated to 80 °C for 24 h, then cooled to room temperature. H_2_SO_4_ 2 M (50 ml) was added, then the organic phase was washed with water, brine and dried. The crude was purified by column chromatography (PE/EtOAc 7:3 as eluent) to afford (1E,4Z,6E)-1-(4-(dimethylamino)phenyl)-5-hydroxy-7-(3-methoxy-4-(prop-2-yn-1-yloxy)phenyl)hepta-1,4,6-trien-3-one (**7**, 70 mg, 19% yield) as a red powder. ^1^H NMR (300 MHz, CDCl_3_) *δ* 7.63 (d, *J* = 15.8 Hz, 1H), 7.57 (d, *J* = 15.8 Hz, 1H), 7.46 (d, *J* = 8.9 Hz, 2H), 7.08 (m, 3H), 6.69 (d, *J* = 8.6 Hz, 2H), 6.50 (d, *J* = 15.8 Hz, 1H), 6.43 (d, *J* = 15.8 Hz, 1H), 5.78 (s, 1H), 4.80 (d, *J* = 2.4 Hz, 2H), 3.92 (s, 3H), 3.03 (s, 6H), 2.53 (t, *J* = 2.3 Hz, 1H); ^13^C NMR (100 MHz, CDCl_3_) *δ* 185.08, 181.34, 151.78, 149.75, 148.45, 141.69, 139.25, 131.06, 130.05, 129.48, 128.32, 127.23, 124.40, 123.80, 122.70, 121.90, 118.92, 117.97, 113.81, 111.91, 110.33, 101.26, 78.12, 76.24, 56.63, 55.93, 40.13; HRESIMS *m*/*z* [M + H]^+^ 404.1859 (calcd for C_25_H_27_NO_4_, 404.1862).

### 4-(2-Azidoethoxy)benzaldehyde (8)

To a stirred solution of 4-hydroxybenzaldehyde (1 g, 8.188 mmol, 1 eq) in DMF (10 ml) K_2_CO_3_ (2.26 g, 16.376 mmol, 2 eq) and 2-bromoethanol (1.16 ml, 16.376 mmol, 2 eq) were added. The reaction was heated at 90 °C for 48 h, then cooled to room temperature. H_2_SO_4_ 2 M (20 ml) was added, then the mixture was extracted with PE/Et_2_O 3:1 (50 ml) and the organic phase was washed with water, brine and dried. The crude was purified by column chromatography (PE/EtOAc 5:5 as eluent) to afford 4-(2-Hydroxyethoxy)benzaldehyde (1.36 g, 100%) as a colorless oil. ^1^H NMR (400 MHz, CDCl_3_) *δ* 9.90 (s, 1H), 7.85 (d, *J* = 9.3 Hz, 2H), 7.03 (d, *J* = 8.7 Hz, 2H), 4.18 (t, *J* = 9.0 Hz, 2H), 4.02 (q, *J* = 9.3 Hz, 2H); ^13^C NMR (100 MHz, CDCl_3_) *δ* 191.07, 163.81, 132.07, 130.05, 114.89, 114.85, 69.62, 61.04; HRESIMS *m*/*z* [M + H]^+^ 166.0705 (calcd for C_9_H_11_O_3_, 166.0708).

A portion of the latter (680 mg, 4.094 mmol, 1 eq) was then dissolved in DCM (10 ml), then TEA (628 ml, 4.503 mmol, 1.1 eq) and methansulfonyl chloride (350 ml, 4.503 mmol, 1.1 eq) were added. The reaction was left at room temperature for 12 h, then quenched with H_2_SO_4_ 2 M (20 ml). The organic phase was then washed with water, brine and dried. The crude was dissolved in DMF (10 ml), then sodium azide (798 mg, 12.382 mmol, 3 eq) and a catalytic amount of NaI were added. The reaction was heated at 65 °C for 12 h, then quenched with brine. The organic phase was dried, and the crude was purified by column chromatography (PE/EtOAc 5:5 as eluent) to afford 4-(2-Azidoethoxy)benzaldehyde (**8**, 712 mg, 91% over two steps) as a yellow oil. ^1^H NMR (400 MHz, CDCl_3_) *δ* = 3.66 (t, *J* = 4.9 Hz, 2H), 4.23 (t, *J* = 4.9 Hz, 2H), 7.04 (d, *J* = 8.8 Hz, 2H), 7.85 (d, *J* = 8.8 Hz, 2H), 9.91 (s, 1H). ^13^C NMR (100 MHz, CDCl_3_) *δ* = 50.0, 67.2, 114.8, 130.5, 132.0, 163.1, 190.7; HRESIMS *m*/*z* [M + H]^+^ 192.0774 (calcd for C_9_H_10_N_3_O_2_, 192.0773).

### Ethyl 2-azidoacetate (9)

To a stirred solution of ethyl 2-bromoacetate (11, 1 ml, 9.017 mmol, 1 eq) in DMF (10 ml), sodium azide (1.759 mg, 27.052 mmol, 3 eq) was added. The reaction was heated at 90 °C for 12 h, then quenched with brine and extracted with PE. The organic phase was dried, and concentration of the solvent under reduced pressure gave ethyl 2-azidoacetate (**9**, 1.16 g, 100%) as a colorless oil. ^1^H NMR (400 MHz, CDCl_3_): *δ* 4.15 (q, *J* = 6.8 Hz, 2H), 3.77 (d, *J* = 1.2 Hz, 2H), 1.20 (td, *J*_1_ = 7.2, *J*_2_ = 1.2 Hz, 3H). ^13^C NMR (100 MHz, CDCl_3_): *δ* 168.1, 61.5, 50.0, 13.8; HRESIMS *m*/*z* [M + H]^+^ 130.0615 (calcd for C_4_H_8_N_3_O_2_, 130.0617).

### General procedure for copper catalyzed 1,3-dipolar cycloaddition: synthesis of Probe **10** as example

To a stirred solution of **6** (50 mg, 0.123 mmol, 1 eq) in *t*-BuOH/H_2_O/CH_3_CN 2:1:1, **8** (56 mg, 0.256 mmol, 2 eq) and a catalytic amount of CuSO_4_ and sodium ascorbate were added. The solution was stirred at room temperature for 24 h, then diluted with brine and extracted with EtOAc. The organic phase was dried and evaporated, then the crude was purified by chromatography over silica gel (PE:EtOAc 3:7 as solvent) to afford **10** (32 mg, 30%) as an orange solid. Probes **11** and **12** were obtained following the same protocol.

**10**: orange powder, 30%. ^1^H NMR (400 MHz, CDCl_3_) *δ* = 9.91 (s, 1H), 7.87–7.83 (m, 3H), 7.63 (d, *J* = 10.4 Hz, 1H), 7.59 (d, *J* = 10.4 Hz, 1H), 7.16–7.07 (m, 4H), 6.98–6.92 (m, 3H), 6.51 (dd, *J*_1_ = 15.8 Hz, *J*_2_ = 2.9 Hz, 2H), 5.84 (s, 1H), 5.37 (s, 2H), 4.83 (t, *J* = 4.7 Hz, 2H), 4.48 (t, *J* = 4.7 Hz, 2H), 3.07 (s, 3H), 2.55 (s, 3H). ^13^C NMR (100 MHz, CDCl_3_) *δ* 190.6, 183.6, 182.7, 162.5, 149.6, 149.3, 147.9, 146.8, 144.0, 140.7, 140.0, 132.0, 130.8, 128.9, 127.6, 124.3, 122.9, 122.5, 122.1, 121.7, 114.8, 114.7, 113.7, 110.4, 109.6, 101.3, 66.4, 62.8, 55.9, 49.7; HRESIMS *m*/*z* [M + H]^+^ 598.21869 (calcd for C_33_H_32_N_3_O_8_, 598.21839).

**11**: brown powder, 33%. ^1^H NMR (400 MHz, CDCl_3_) *δ* = 7.83 (s, 1H), 7.60 (dd, *J*_1_ = 15.7 Hz, *J*_2_ = 4.5 Hz, 2H), 7.14–7.07 (m, 4H), 6.95 (d, *J* = 8.2 Hz, 1H), 6.50 (dd, *J*_1_ = 15.7 Hz, *J*_2_ = 2.8 Hz, 2H), 5.83 (s, 1H), 5.38 (s, 2H), 5,17 (s, 2H), 4.28 (q, *J* = 7.1, 2H), 3.69 (s, 3H), 3.92 (s, 3H), 1.31 (t, *J* = 7.1 Hz, 3H). ^13^C NMR (100 MHz, CDCl_3_) *δ* 183.6, 182.9, 166.0, 149.6, 149.4, 147.9, 146.8, 144.2, 140.7, 140.1, 128.9, 127.6, 124.5, 124.5, 122.9, 122.4, 122.3, 121.7, 114.8, 113.8, 110.5, 109.6, 101.3, 62.8, 62.5, 55.9, 51.0, 14.0; HRESIMS *m*/*z* [M + H]^+^ 536.20245 (calcd for C_28_H_30_N_3_O_8_, 536.202741).

**12**: red powder, 31%. ^1^H NMR (400 MHz, CDCl_3_) *δ* = 9.91 (s, 1H), 7.84–7.86 (m, 2H), 7.67–7.58 (m, 3H), 7.20–7.03 (m, 3H), 6.97 (d, *J* = 8.7 Hz, 2H), 6.57 (d, *J* = 15.8 Hz, 2H), 6.52 (d, *J* = 15.8 Hz, 2H), 5.85 (s, 1H), 5.37 (s, 1H), 4.83 (t, *J* = 4.9 Hz, 2H), 4.48 (t, *J* = 4.9 Hz, 2H), 2.33 (s, 3H), 1.24 (s, 6H). ^13^C NMR (100 MHz, CDCl_3_) *δ* ^13^C NMR (100 MHz, CDCl_3_) *δ* 190.6, 162.5, 149.6, 132.0, 130.8, 129.8, 124.2, 122.5, 114.7, 113.7, 110.4, 81.7, 66.4, 62.9, 56.0, 49.6, 29.7; HRESIMS *m*/*z* [M + H]^+^ 628.23096 (calcd for C_34_H_35_N_4_O_6_, 628.22896).

### General procedure for diethylphosphate derivative: synthesis of probe 13 as example

To stirred solution of **10** (150 mg, 0.251 mmol, 1 eq) in dry DCM (10 ml), TEA (105 ml, 0.753 mmol, 3 eq) and diethylchlorophosphate (54 ml, 0.377 mmol, 1.5 eq) were added. The reaction was stirred at room temperature for 24 h, then quenched with H_2_SO_4_ 2 M. The organic phase was then washed with brine and dried. The crude was purified by chromatography over silica (EtOAC as eluent) to afford Probe **13** (47 mg, 23%) as orange solid. Probe **14** was obtained following the same protocol.

**13**: orange solid, 23%. ^1^H NMR (400 MHz, CDCl_3_) *δ* = 9.90 (s, 1H), 7.83–7.79 (m, 3H), 7.61 (d, *J* = 15.7 Hz, 2H), 7.35–6.95 (m, 7H), 6.54 (m, 2H), 5.86 (s, 1H), 5.37 (s, 2H), 4.83 (bt, 2H), 4.47 (bt, 2H), 4.30 (m, 4H) 3.95 (s, 3H), 3.93 (s, 3H), 1.39 (t, *J* = 6.89 Hz, 6H). ^13^C NMR (100 MHz, CDCl_3_) *δ* 190.6, 183.9, 182.3, 162.5, 150.9, 150.9, 149.6, 149.4, 143.9, 141.3, 140.5, 139.5, 132.8, 132.0, 130.7, 128.8, 124.3, 124.0, 122.5, 122.3, 121.6, 121.1, 114.7, 113.7, 111.7, 110.5, 101.6, 66.4, 64.7, 64.7, 62.8, 56.0, 49.7, 16.1, 16.0; HRESIMS *m*/*z* [M + H]^+^ 734.24738 (calcd for C_37_H_41_N_3_O_11_P, 734.24732).

**14**: brown solid, 76%. ^1^H NMR (400 MHz, CDCl_3_) *δ* 7.83 (s, 1H), 7.61 (d, *J* = 6.7 Hz, 1H), 7.57 (d, *J* = 6.7 Hz, 1H), 7.32 (d, *J* = 7.9 Hz, 1H),7.13–7.08 (m, 4H), 6.57–6.49 (m, 2H), 5.85 (s, 3H), 5.36 (s, 2H), 5.16 (s, 2H), 4.30–4.19 (m, 6H), 3.92 (s, 3H), 3.91 (s, 3H), 1.38 (t, *J* = 7.0 Hz, 3H). 1.30 (t, *J* = 7.0 Hz, 3H). ^13^C NMR (100 MHz, CDCl_3_) *δ* 184.0, 182.2, 166.0, 150.8, 149.7, 149.5, 144.1, 141.3, 140.6, 139.4, 132.8, 128.7, 124.6, 124.0, 122.3, 121.5, 121.0, 113.7, 111.7, 110.5, 101.6, 64.7, 62.8, 62.5, 56.0, 51.0, 29.7, 16.1, 14.0; HRESIMS *m*/*z* [M + H]^+^ 672.23120 (calcd for C_32_H_39_N_3_O_11_P, 672.23167).

### General procedure for copper catalyzed 1,3-dipolar cycloaddition: synthesis of Probe **10** as example

To a stirred solution of **6** (50 mg, 0.123 mmol, 1 eq) in *t*-BuOH/H_2_O/CH_3_CN 2:1:1, **8** (56 mg, 0.256 mmol, 2 eq) and a catalytic amount of CuSO_4_ and sodium ascorbate were added. The solution was stirred at room temperature for 24 h, then diluted with brine and extracted with EtOAc. The organic phase was dried and evaporated, then the crude was purified by chromatography over silica gel (PE:EtOAc 3:7 as solvent) to afford **10** (32 mg, 30%) as an orange solid. Probes **11** and **12** were obtained following the same protocol.

**10**: orange powder, 30%. ^1^H NMR (400 MHz, CDCl_3_) *δ* = 9.91 (s, 1H), 7.87–7.83 (m, 3H), 7.63 (d, *J* = 10.4 Hz, 1H), 7.59 (d, *J* = 10.4 Hz, 1H), 7.16–7.07 (m, 4H), 6.98–6.92 (m, 3H), 6.51 (dd, *J*_1_ = 15.8 Hz, *J*_2_ = 2.9 Hz, 2H), 5.84 (s, 1H), 5.37 (s, 2H), 4.83 (t, *J* = 4.7 Hz, 2H), 4.48 (t, *J* = 4.7 Hz, 2H), 3.07 (s, 3H), 2.55 (s, 3H). ^13^C NMR (100 MHz, CDCl_3_) *δ* 190.6, 183.6, 182.7, 162.5, 149.6, 149.3, 147.9, 146.8, 144.0, 140.7, 140.0, 132.0, 130.8, 128.9, 127.6, 124.3, 122.9, 122.5, 122.1, 121.7, 114.8, 114.7, 113.7, 110.4, 109.6, 101.3, 66.4, 62.8, 55.9, 49.7; HRESIMS *m*/*z* [M + H]^+^ 598.21869 (calcd for C_33_H_32_N_3_O_8_, 598.21839).

**11**: brown powder, 33%. ^1^H NMR (400 MHz, CDCl_3_) *δ* = 7.83 (s, 1H), 7.60 (dd, *J*_1_ = 15.7 Hz, *J*_2_ = 4.5 Hz, 2H), 7.14–7.07 (m, 4H), 6.95 (d, *J* = 8.2 Hz, 1H), 6.50 (dd, *J*_1_ = 15.7 Hz, *J*_2_ = 2.8 Hz, 2H), 5.83 (s, 1H), 5.38 (s, 2H), 5,17 (s, 2H), 4.28 (q, *J* = 7.1, 2H), 3.69 (s, 3H), 3.92 (s, 3H), 1.31 (t, *J* = 7.1 Hz, 3H). ^13^C NMR (100 MHz, CDCl_3_) *δ* 183.6, 182.9, 166.0, 149.6, 149.4, 147.9, 146.8, 144.2, 140.7, 140.1, 128.9, 127.6, 124.5, 124.5, 122.9, 122.4, 122.3, 121.7, 114.8, 113.8, 110.5, 109.6, 101.3, 62.8, 62.5, 55.9, 51.0, 14.0; HRESIMS *m*/*z* [M + H]^+^ 536.20245 (calcd for C_28_H_30_N_3_O_8_, 536.202741).

**12**: red powder, 31%. ^1^H NMR (400 MHz, CDCl_3_) *δ* = 9.91 (s, 1H), 7.84–7.86 (m, 2H), 7.67–7.58 (m, 3H), 7.20–7.03 (m, 3H), 6.97 (d, *J* = 8.7 Hz, 2H), 6.57 (d, *J* = 15.8 Hz, 2H), 6.52 (d, *J* = 15.8 Hz, 2H), 5.85 (s, 1H), 5.37 (s, 1H), 4.83 (t, *J* = 4.9 Hz, 2H), 4.48 (t, *J* = 4.9 Hz, 2H), 2.33 (s, 3H), 1.24 (s, 6H). ^13^C NMR (100 MHz, CDCl_3_) *δ* ^13^C NMR (100 MHz, CDCl_3_) *δ* 190.6, 162.5, 149.6, 132.0, 130.8, 129.8, 124.2, 122.5, 114.7, 113.7, 110.4, 81.7, 66.4, 62.9, 56.0, 49.6, 29.7; HRESIMS *m*/*z* [M + H]^+^ 628.23096 (calcd for C_34_H_35_N_4_O_6_, 628.22896).

### General procedure for diethylphosphate derivative: synthesis of probe 13 as example

To stirred solution of **10** (150 mg, 0.251 mmol, 1 eq) in dry DCM (10 ml), TEA (105 ml, 0.753 mmol, 3 eq) and diethylchlorophosphate (54 ml, 0.377 mmol, 1.5 eq) were added. The reaction was stirred at room temperature for 24 h, then quenched with H_2_SO_4_ 2 M. The organic phase was then washed with brine and dried. The crude was purified by chromatography over silica (EtOAC as eluent) to afford Probe **13** (47 mg, 23%) as orange solid. Probe **14** was obtained following the same protocol.

**13**: orange solid, 23%. ^1^H NMR (400 MHz, CDCl_3_) *δ* = 9.90 (s, 1H), 7.83–7.79 (m, 3H), 7.61 (d, *J* = 15.7 Hz, 2H), 7.35–6.95 (m, 7H), 6.54 (m, 2H), 5.86 (s, 1H), 5.37 (s, 2H), 4.83 (bt, 2H), 4.47 (bt, 2H), 4.30 (m, 4H) 3.95 (s, 3H), 3.93 (s, 3H), 1.39 (t, *J* = 6.89 Hz, 6H). ^13^C NMR (100 MHz, CDCl_3_) *δ* 190.6, 183.9, 182.3, 162.5, 150.9, 150.9, 149.6, 149.4, 143.9, 141.3, 140.5, 139.5, 132.8, 132.0, 130.7, 128.8, 124.3, 124.0, 122.5, 122.3, 121.6, 121.1, 114.7, 113.7, 111.7, 110.5, 101.6, 66.4, 64.7, 64.7, 62.8, 56.0, 49.7, 16.1, 16.0; HRESIMS *m*/*z* [M + H]^+^ 734.24738 (calcd for C_37_H_41_N_3_O_11_P, 734.24732).

**14**: brown solid, 76%. ^1^H NMR (400 MHz, CDCl_3_) *δ* 7.83 (s, 1H), 7.61 (d, *J* = 6.7 Hz, 1H), 7.57 (d, *J* = 6.7 Hz, 1H), 7.32 (d, *J* = 7.9 Hz, 1H), 7.13–7.08 (m, 4H), 6.57–6.49 (m, 2H), 5.85 (s, 3H), 5.36 (s, 2H), 5.16 (s, 2H), 4.30–4.19 (m, 6H), 3.92 (s, 3H), 3.91 (s, 3H), 1.38 (t, *J* = 7.0 Hz, 3H). 1.30 (t, *J* = 7.0 Hz, 3H). ^13^C NMR (100 MHz, CDCl_3_) *δ* 184.0, 182.2, 166.0, 150.8, 149.7, 149.5, 144.1, 141.3, 140.6, 139.4, 132.8, 128.7, 124.6, 124.0, 122.3, 121.5, 121.0, 113.7, 111.7, 110.5, 101.6, 64.7, 62.8, 62.5, 56.0, 51.0, 29.7, 16.1, 14.0; HRESIMS *m*/*z* [M + H]^+^ 672.23120 (calcd for C_32_H_39_N_3_O_11_P, 672.23167).

### Expression and purification of the recombinant human aldehyde dehydrogenases 1A subfamily

A common experimental protocol has been developed with the aim of obtaining pure human ALDH1A1, 1A2 and 1A3 at a high yield, as already described^23^. Briefly, *E. coli* BL21 (DE3) were transformed with the full-length expression vector of each isoform and seeded onto 2xTY agar plates containing 50 μg/ml ampicillin for ALDH1A1 and ALDH1A3 subtypes, and 50 μg/ml kanamycin for ALDH1A2. Petri plates were incubated for the overnight growth at 37 °C. The following day, colonies were scraped and used to inoculate 1 l of 2xTY liquid medium, which was previously added with 50 μg/ml ampicillin for ALDH1A1 and ALDH1A3, and 50 μg/ml kanamycin for ALDH1A2. Flasks were put under shaking at 37 °C and, once OD_600_ = 0.6–0.8 was reached, the temperature was shifted to 20 °C to induce the recombinant protein production. The induced cells were collected by centrifugation and stored at −80 °C. The harvested pellet was thawed and resuspended in lysis buffer (50 mM Na2HPO4, 300 mM NaCl, 1 mM β-mercaptoethanol, 20 mM imidazole, pH 7.5) with 1 μl per 80 ml of lysis buffer of benzonase nuclease (250 U/μl). *E. coli* BL21 (DE3) cells were disrupted using a French Press system, three times at 1.5 Kbar, adding 100 μl per 40 ml of lysis buffer of a Protease inhibitor cocktail from SIGMA. To obtain the clarified cell lysate, the cell debris was removed by centrifugation at 18,000 rpm for 50 min. The recombinant proteins were purified by a His-tag affinity chromatography followed by size-exclusion chromatography, using an AKTA FPLC system at 4 °C. To better evaluate the purity and homogeneity of the protein after each purification step, eluted fractions were analyzed by SDS-PAGE. The final protein concentration was determined through the Bradford protein assay. In the first purification step, the collected supernatant was loaded on a Qiagen Ni-NTA Superflow 5 ml cartridge that was previously equilibrated with 10 column volumes of lysis buffer. The Ni-NTA cartridge was washed with 15 column volumes of 50 mM Na2HPO4, 300 mM NaCl, 1 mM β-mercaptoethanol, 50 mM imidazole, pH 7.5, until the absorbance at 280 nm returned to the baseline. The recombinant hALDH1A was eluted with 50 mM Na2HPO4, 300 mM NaCl, 1 mM β-mercaptoethanol, 250 mM imidazole, pH 8, by applying a linear gradient in 10 column volumes. Eluted fractions were pooled and concentrated to 5 ml with Merck Millipore Amicon Ultra-15 30 kDa and loaded on a HiLoad 16/600 Superdex 200 pg column on AKTA FPLC system. Elution buffer contained 20 mM Tris HCl pH 8.0, 150 mM KCl, 1 mM β-mercaptoethanol, and a flow rate of 1 ml/min was applied. By means of this procedure, 20 mg of pure and active human ALDH1A1, ALDH1A2 and ALDH1A3 were obtained, stocked at −80° and later used for biochemical analysis.

### Absorbance, emission and excitation wavelengths evaluation

Fluorescent compounds were analyzed on a Tecan Spark to evaluate the correct parameters to further set the biochemical characterizations. The absorbance was evaluated in a range from 300 to 700 nm, with a wavelength step size of 5 nm. Based on the absorbance values, we settled a 3D fluorescence emission scan to evaluate the excitation and emission peaks. The analysis was conducted using the same buffer mix, as already described. The excitation range was set between 390 and 450 nm for both molecules, with a step size of 5 nm. The emission range was set between 485 and 700 nm for both molecules, with a step size of 5 nm. All these characterizations were performed on a Tecan Spark using Greiner Bio-One 96-UV-Transparent Microplates and the tests were carried out using a total volume of 100 μl for each well.

### Chemical stability and cross reactions with biomolecules assay

Based on the values obtained by the 3D analysis, a wide series of biomolecules directly used in the biochemical experiment, both in vitro and in vivo, and listed in Table 1, were tested in complex with a fixed concentration of 10 μM of both probes, to measure any possible cross reaction signal.

**Table. 1:** List of a several biomolecules directly used in the biochemical experiment, both in vitro and in vivo.

Chemical species	Assay concentration
CONTROL (only SEC buffer)	See material and methods
Tris HCl pH = 8.0	20 mM
Tris HCl pH = 8.5	20 mM
Hepes Na pH = 7.0	20 mM
Hepes Na pH = 7.5	20 mM
H_2_O_2_	100 μM
FBS	0.1% v/v
Trypsine	0.05 mg/ml
NaOH	100 μM
FeCl_2_	100 μM
β-mercaptoethanol	1 mM
Cysteine	100 μM
Glycine	100 μM
Glutamate	100 μM
Lysine	100 μM
Tryptophane	100 μM
Tyrosine	100 μM
NaCl	150 mM
KCl	150 mM
MgCl_2_	2.5 mM
ZnSO_4_	100 μM
NAD^+^	500 μM
DMSO	15%
Probe 10/11 diluted in SEC buffer	Probe 10/11 = 10 μM
Probe 10/11 diluted in SEC buffer + ALDH1A1	ALDH1A1 = 100 μM
Probe 10/11 diluted in SEC buffer + ALDH1A2	ALDH1A2 = 100 μM
Probe 10/11 diluted in SEC buffer + ALDH1A3	ALDH1A3 = 100 μM

### *K*_d_ evaluation of the probes in complex with human ALDH1A isoforms

To evaluate the *K*_d_ constant between the two probes and the isoenzymes ALDH1A1, ALDH1A2 and ALDH1A3A we performed a fluorescence emission assay. We used a Tecan Spark with Greiner Bio-One 96-UV-Transparent Microplates. A single reaction was performed in a total volume of 100 μl per well containing 20 mM Tris HCl pH 8.0, 1 mM β-mercaptoethanol, 150 mM KCl, 500 μM NAD^+^ and 10 μM fluorescent probe with a 5% DMSO final concentration, in the presence of different ALDH1As concentrations, from 100 μM to 1.1719 μM. Each reaction mix was preincubated for 10 min at 25 °C before the analysis. The obtained raw data were analyzed using GraphPad to calculate *K*_d_ values.

### *K*_M_ evaluation of probes in complex with the human ALDH1As isoforms

The catalytic activity of the ALDH1As isoenzymes in complex with probe **10** was tested on a Tecan Sunrise 96 Multiplate Reader with Greiner Bio-One 96-UV-Transparent Microplates. The analysis was performed in triplicate in a total volume of 100 μl per well containing 20 mM Tris HCl pH 8.0, 1 mM β-mercaptoethanol, 150 mM KCl, 500 μM NAD^+^, 1.41 μM DMSO, 2.8 μM hALDH1As and Probe **10** was tested as substrate at different concentrations, from 300 μM to 3.125 μM. Each reaction mix was preincubated for 10 min at 25 °C before the analysis. The catalytic activity was measured by monitoring the absorbance at 340 nm (εNADH = 6220 M^−1^ cm^−1^) for 12 h at 25 °C and the inhibitory parameters were calculated by processing the raw data on GraphPad.

### IC_50_ and *K*_i_ evaluation of probes on the ALDH1A isoforms

Initially, the inhibitory activities of our fluorescent probes were screened at a fixed concentration of 50 μM using a Tecan Sunrise 96 Multiplate Reader with Greiner Bio-One 96-UV-Transparent Microplates. The analysis was performed in triplicate in a total volume of 100 μl per well containing 20 mM Tris HCl pH 8.0, 1 mM β-mercaptoethanol, 150 mM KCl, 500 μM NAD^+^, 1.41 μM DMSO, 2.8 μM hALDH1A and 20 mM acetaldehyde. Afterwards, the inhibitory potency of the fluorescent probes was further investigated, evaluating the IC_50_ parameter. The enzymatic inhibition assays were performed in triplicate in a total volume of 100 μl per well containing 20 mM Tris HCl pH 8.0, 1 mM β-mercaptoethanol, 150 mM KCl, 500 μM NAD^+^, 2.8 μM hALDH1A and 20 mM acetaldehyde in the presence of different probe concentrations, from 200 μM to 1.5625 μM. The DMSO final concentration allowed was up to 15%. Only for ALDH1A3 in complex with Probe **10**, the *K*_i_ affinity parameter was calculated by performing the analysis in triplicate in a total volume of 100 μl per well containing 20 mM Tris HCl pH 8.0, 1 mM β-mercaptoethanol, 150 mM KCl, 500 μM NAD^+^ and 2.8 μM ALDH1As. Due to chemical nature of Probe **10** and Probe **11**, we set up the enzymatic assay assuming a competitive inhibition using different compounds concentration, from 200 μM to 1 μM, and different acetaldehyde concentrations, from 20 mM to 2.5 mM. Each reaction mix was preincubated for 10 min at 25 °C before the analysis. The catalytic activity was measured by monitoring the absorbance at 340 nm (εNADH = 6220 M^−^^1^ cm^−1^) for 30 min at 25 °C and the inhibitory parameters were calculated by processing the raw data on SigmaPlot^53^.

### LC-HRMS analysis of ALDH1As isoenzyme activity in the presence of Probe 10 or Probe 11

These analyses were carried out by using UPLC-HRMS procedure with Vanquish UPLC system coupled to a Thermo Scientific Q-Exactive Plus, operating in negative electrospray ionization mode. The chromatographic separation was carried out using a Phenomenex Synergi 4 µ Polar-RP 80 A (150 × 2 mm) equipped with a Phenomenex Polar-RP (4 mm × 2 mm) security guard column. The mobile phase consisted of A: acetonitrile 0.1% formic acid and B: 0.1% formic acid. We used a linear elution gradient, starting from 80% of A to 5% A in 3 min, 3 min at 5% A, an equilibration of 4 min for a total run time of 10.00 min, at 300 µl/min flow rate. The autosampler temperature was set at 15 °C, the injection volume was 5 µl and column was maintained at 40 °C. The heated electrospray ionization source was used, with a spray voltage of 3200 kV, with a capillary temperature of 300 °C, a heater temperature of 350 °C, a sheat gas flow of 45 arbitrary units (AU), an auxiliary gas flow of 10 AU and a sweep gas flow of 0 AU. During the Parallel Reaction Monitoring acquisition in negative ion mode, the instrument operated at 17,500 resolution in presence of the inclusion list (*m*/*z* = 596.20384 and *m*/*z* = 612.19875 for Probe **10** and Probe **10-COOH** respectively; *m*/*z* = 534.18819 and *m*/*z* = 506.156888 for Probe **11** and Probe **11-COOH** respectively) with an automatic gain control target of 2 × 10^5^ charges and a maximum injection time of 100 ms. Before injecting the samples, the blanks in the list were analyzed and every biological sample was analyzed in duplicate. The biological samples analyzed were as follow: (1) Enzymatic assay Buffer; (2) Enzymatic assay Buffer + NAD^+^; (3) Enzymatic assay Buffer + Probe **10** or Probe **11**; (4) Enzymatic assay Buffer + ALDH1A1; (5) Enzymatic assay Buffer + ALDH1A2; (6) Enzymatic assay Buffer + ALDH1A3; (7) Enzymatic assay Buffer + ALDH1A1 + Probe **10** or Probe **11**; (8) Enzymatic assay Buffer + ALDH1A2 + Probe **10** or Probe **11**; (9) Enzymatic assay Buffer + ALDH1A3 + Probe **10** or Probe **11**. All samples were diluted 1:1 with ACN, then 1:100 in ACN-Water 1:1 prior the LC-HRMS analysis.

### Cell culture

U87MG human glioblastoma, and 4T1 murine mammary carcinoma cell lines were cultured in Minimum Essential Medium Eagle (Sigma-Aldrich). The HEK293T human embryonic kidney cell line and GL261 high grade glioma cells were cultured in Dulbecco’s Modified Eagle’s Medium (DMEM, Sigma-Aldrich). All these cell lines with the exception of GL261 were purchased from ATCC. Media were supplemented with 10% fetal bovine serum (Gibco), 2 mg/ml glutamine, 10 U/ml penicillin and 100 g/ml streptomycin (Sigma-Aldrich). Normal human fetal astrocytes were kindly provided by Eleonora Aronica’s lab in Amsterdam and cultured in DMEM + F10 medium. Cells were maintained in a controlled atmosphere of 5% CO_2_ with humidity at 37 °C. Cells were detached from plates by trypsin-EDTA (Sigma-Aldrich). Patient-derived glioblastoma cells (#3054 and #3060) were acquired from the Human Glioblastoma Cell Culture resource (www.hgcc.se) at the Department of Immunology, Genetics and Pathology, Uppsala University, Uppsala, Sweden and cultured as previously reported^54^. Briefly, cells were cultured in 1:1 DMEM/F12:Neurobasal, in presence of 100X of N2, 50X of B27 (Gibco, Thermo Fisher Scientific), 10 ng/ml human FGF2 and 10 ng/ml human EGF (PeproTech).

### Fluorescent microscopy

In total, 20,000 cells/ml of U87MG, HEK293T, hASTRO and 4T1 were seeded onto glass cover slips in 24-well plates. The cells incubated at 37 °C in a 5% CO_2_ atmosphere overnight. Cells were washed with 1 ml of PBS buffer two times, then a 10 μM solution of probes was added to each well for 2 h. For nuclei staining, 1 µg/ml of DAPI was added the last 15 min of treatment. The cover slips were removed and washed with PBS buffer, and fixed with 0.5 ml of 4% formaldehyde solution for 10 min. The cover slips were washed with 1 ml of PBS, then slides were prepared using Mounting Media (Merck Life Science). Fluorescence images were acquired using a Leica THUNDER Imager 3D Live Cell (Leica Microsystems, Wetzlar, Germany) microscope equipped with an S Fluor 40×/1.3 objective using the LAS X software.

### Flow cytometry

U87MG, 3054, 3060, HEK293T, hASTRO and 4T1 cells were resuspended in PBS to a final concentration of 10^6^ cells/ml. Each sample was then resuspended in probe **10** and probe **11** solutions (1 µM, in ALDEFLUOR assay buffer, STEMCELL). Triplicate samples were prepared for each dye. Cells were pre-treated with DEAB for 15 min and then incubated with the probes for 30 min at room temperature, with rocking to prevent cell clumping and ensure an even dye distribution. At the end of the incubation period, cells were harvested by centrifugation at 1000 rpm for 5 min at 4 °C. The probe solutions were removed and each sample was resuspended in the ALDEFLUOR assay buffer. The samples were immediately placed in ice until analysis. The samples were analyzed by a S3e Cell Sorter (Bio-Rad).

GL261 cells were incubated, with probe **10** or **11**, for 1 h and after three rinses in PBS, cells were detached by trypsin treatment and visualized without fixation through a rMarkII flow cytometer (Amnis, Luminex Corporation, Austin, TX, USA) as previously described^55^. Data were collected using Inspire software (Amnis, version 2.0) with the following parameters: 10,000 images per sample, 488 nm laser (25 and 100 mW) to excite the probes, 785 nm laser used to provide a side scatter signal and measurement of SpeedBeads (Amnis, Luminex Corporation, Austin, TX, USA), 830 nm laser used for internal bead calibration of core flow speed and focus, ×60 objective, in low-speed flow. Data were further analyzed by Ideas software (Amnis, version 6.1).

### Cell viability

In total, 10 × 10^5^ U87MG human glioblastoma, patient-derived glioma cells 3054 and 3060, HEK293T human embryonic kidney, 4T1 murine mammary carcinoma, human fetal astrocytes cell lines were plated in their respective medium and treated for 72 h with our fluorescent probe **10** and **11**, solubilized in DMSO in a final concentration of 10%. We treated controls with DMSO in the same final concentration and we compared the viability of each treatment to controls. The viability of cells was measured using the 3-(4,5-dimethylthiazol-2-yl)-2,5-diphenyltetrazolium assay (MTT assay)^23^.

### Animal experiments

We used in vitro and in vivo cells from a mouse high grade glioma cell line, GL261^56^. Briefly, ten 2- to 3-month-old female C57BlC mice were stereotactically implanted under deep general anesthesia (isoflurane supplemented with nitrous oxide) with 1 × 10^5^ GL261 glioblastoma cells. The cells were stereotactically inoculated through a burr hole by a Hamilton syringe into the left striatum (coordinates: 1 mm anteroposterior and 1 mm lateral from bregma, at a depth of 3 mm). All experimental procedures were conducted in accordance with the European Communities Council Directive of the 24th of November 1986 (86/609 EEC), with the Recommendation 18/06/2007, Dir. 2010/63/UE and with the Italian law for care and use of experimental animals (DL116/92) and were approved by the Italian Ministry of Health (prot. E669C.15) and by the Bioethical Committee of the University of Turin. All animals were housed under a 12-h light-dark cycle in an environmentally controlled room. All experiments were designed to minimize the numbers of animals used and their discomfort. In each experiment, animals with tumors were allocated to two groups and i.p. injected with probe **10** (18 μg) or probe **11** (5 μg). All drugs were administered intraperitoneally 6 days after tumor implantation and the animals were euthanized under deep anesthesia 6 h after the i.p. injection of the probes. Briefly, they were transcardially perfused under deep anesthesia (ketamine 100 mg/mg, Ketavet, Bayern, Leverkusen, Germany; xylazine 5 mg/kg, Rompun, Bayern, Leverkusen, Germany) with 4% paraformaldehyde in 0.12 M phosphate buffer, pH 7.2 to 7.4. The brains were dissected and cut into 50 μm thick cryostat coronal sections. Sections were incubated and counterstained with 4′, 6-diamidino-2- phenylindole (DAPI). After processing, sections were mounted on microscope slides with Tris-glycerol supplemented with 10% Mowiol (Calbiochem). Quantitative and phenotypic evaluations were made on the images acquired with a Leica TCS SP5 confocal microscope. Fiji (http://fiji.sc/Image_Stitching), Inkscape (http://inkscape.org), and Photoshop CS6 (Adobe Inc. https://www.adobe.com) were used to assemble all figures.

### Reporting summary

Further information on research design is available in the Nature Research Reporting Summary linked to this article.

## Supplementary information

Supplementary Information

Description of Additional Supplementary Files

Supplementary Data 1

Reporting Summary

## Data Availability

All data supporting the findings of this study are available within the paper and its Supplementary Information files. The source data behind the graphs can be found in Supplementary Data 1.
