# Supplementary material for: Curcumin-based-fluorescent probes targeting ALDH1A3 as a promising tool for glioblastoma precision surgery and early diagnosis
Source: Commun Biol. 2022 Sep 1;5:895. doi: 10.1038/s42003-022-03834-7 (PMC9437101; doi:10.1038/s42003-022-03834-7)

Curcumin-based-fluorescent probes targeting ALDH1A3 as a promising tool for glioblastoma precision surgery and early diagnosis

Edoardo L. M. Gelardi[a,1], Diego Caprioglio[a,1], Giorgia Colombo[a], Erika Del Grosso[a], Daniele Mazzoletti[a], Daiana Mattoteia[a], Stefano Salamone[a], Davide M. Ferraris[a,b], Eleonora Aronica[c,d], Giulia Nato[e,f], Annalisa Buffo[e,g], Menico Rizzi[a], Lorenzo Magrassi[h,i], Alberto Minassi[a,j] * & Silvia Garavaglia[a,*].

[a] Department of Scienze del Farmaco, University of Piemonte Orientale, Via Bovio, 6, Novara, 28100, Italy

[b] IXTAL srl, via Bovio 6, 28100, Novara, Italy

[c] Department of (Neuro)Pathology, Amsterdam UMC, University of Amsterdam, Amsterdam, The Netherlands

[d] Stichting Epilepsie Instellingen Nederland (SEIN), Heemstede, The Netherlands

[e] Department of Life Sciences and System Biology University of Turin, Via accademia Albertina 13,Turin, Italy

[f] Neuroscience Institute Cavalieri Ottolenghi (NICO), 10043 Orbassano, Torino, Italy.

[g] Department of Neuroscience Rita Levi Montalcini, University of Turin, Via Cherasco 15, Torino, Italy.

[h] Neurosurgery, Department of Clinical, Surgical, Diagnostic and Pediatric Science, University of Pavia, Foundation IRCCS Policlinico San Matteo, Pavia, 27100, Italy.

[i] Istituto Di Genetica Molecolare IGM-CNR, via Abbiategrasso 207, 27100 Pavia, Italy.

[j] PlantaChem srls, via Canobio 4/6, 28100 Novara, Italy

Correspondence: silvia.garavaglia@uniupo.it; alberto.minassi@uniupo.it

[1] These authors contributed equally to the work

Table of Contents

Supplementary Figure S1: LC-HRMS Characterization of synthesized probes.

Supplementary Figure S2: LC-HRMS analysis for the determination of interaction of probes with different isoenzyme of ALDH1A.

Supplementary Figure S3: Biochemical characterization of the interaction of probe 11 with human ALDH1A3.

Supplementary Figure S4: Flow cytometry analysis of Probe 10 on different cell lines.

Supplementary Figure S5: Probe 11 is selective on U87 ALDH1A3 positive cells.

Supplementary Figure S6: *In vitro* experiments showing that ALDH1A3 is the most expressed isoform in GL261 murine glioma cells and Probe 11 enter and label the same cells.

Supplementary Figure S1. LC-HRMS Characterization of synthesized probes.

Data were obtained by Thermo Fisher Q-Exactive Plus equipped with an Orbitrap (ion trap) mass analyzer

10

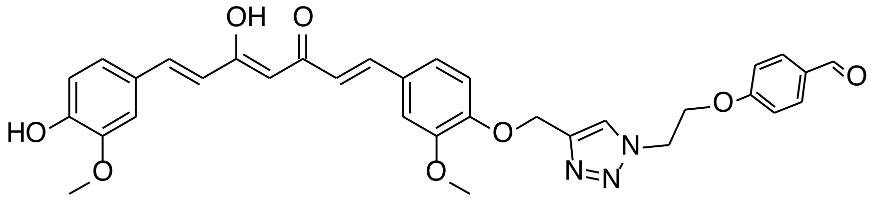

Molecular Formula:	$C_{33}H_{31}N_3O_8$
Formula Weight:	597.61454
[M+H]+:	598.218391 Da
[M-H]-:	596.203839 Da

$[M+H]^+$ Obt. 598.21869 Calc. 598.21839 ppm 0.50

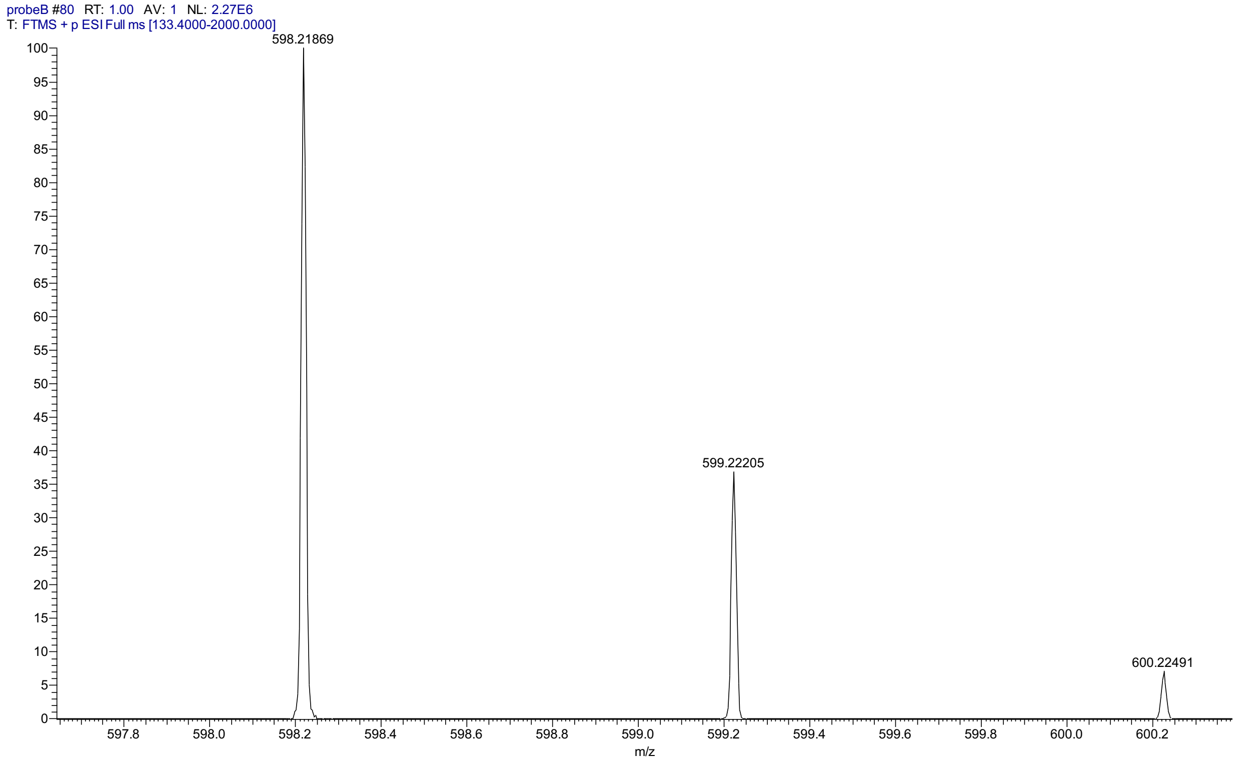

[M-H]⁻ Obt. 596.20432 Calc. 596.20384 ppm 0.64

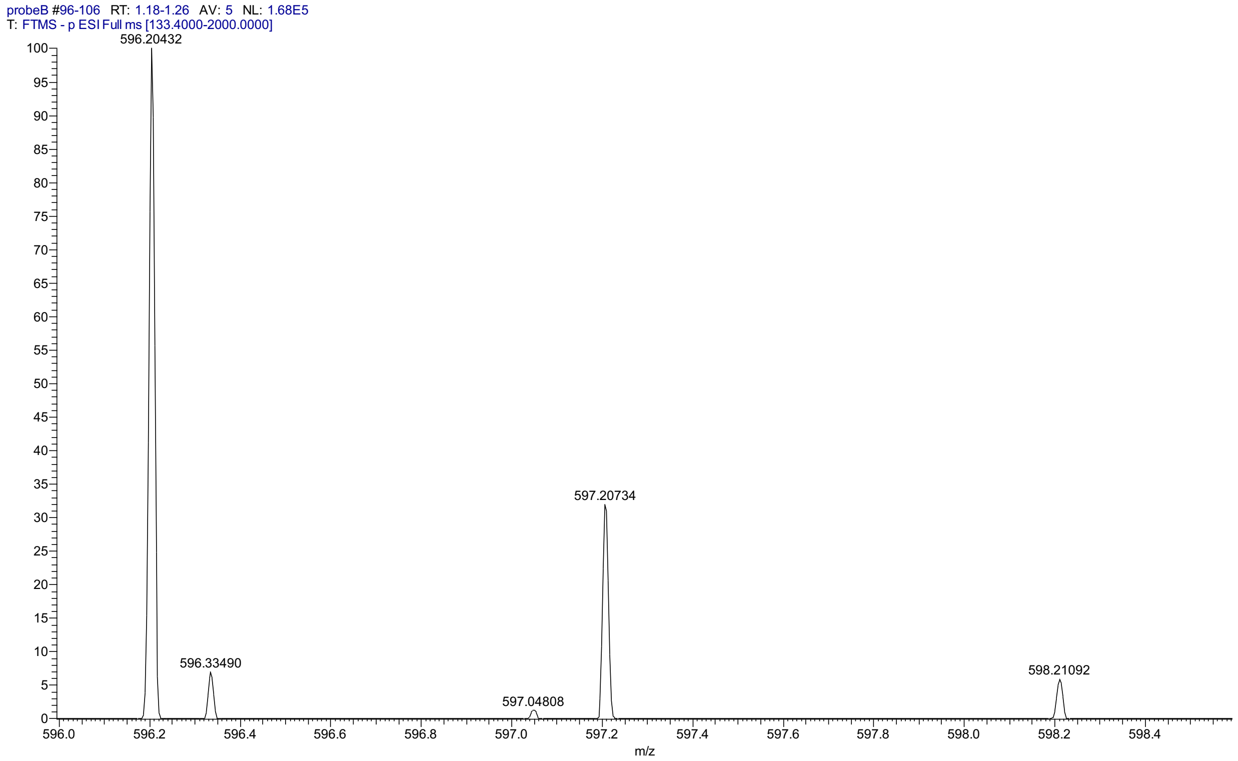

11

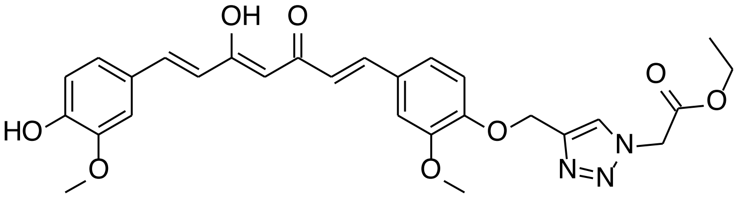

Molecular Formula: $C_{28}H_{29}N_3O_8$
Formula Weight: 535.54516
[M+H]+: 536.202741 Da
[M-H]-: 534.188188 Da

[M+H]⁺ Obt. 536.20245 Calc. 536.202741 ppm 0.54

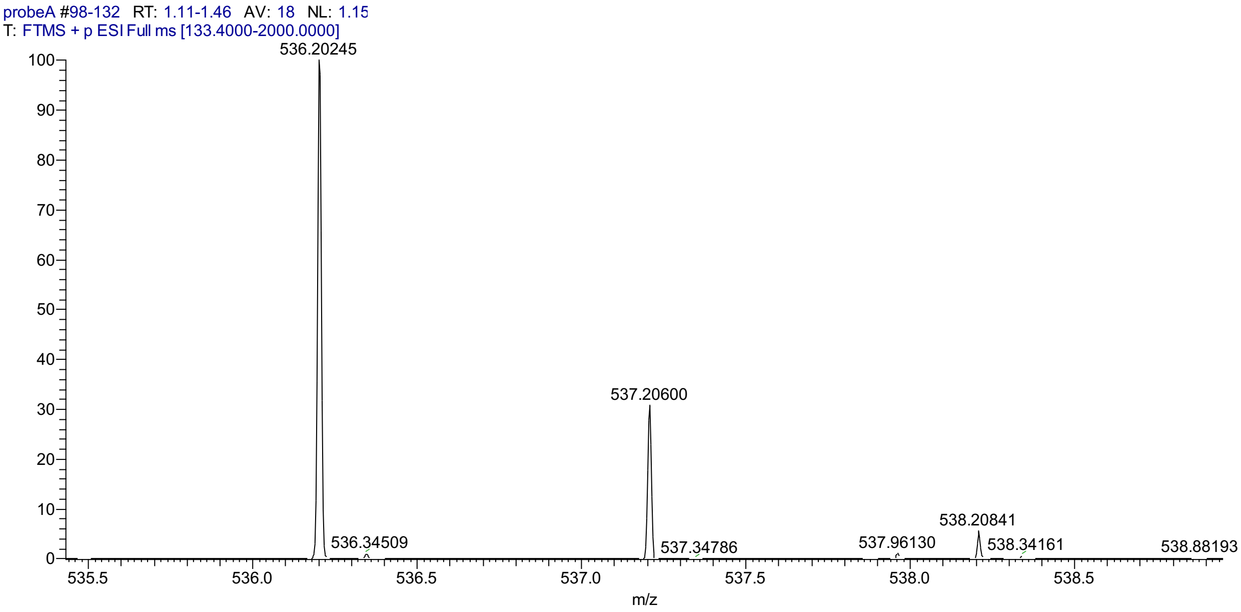

12

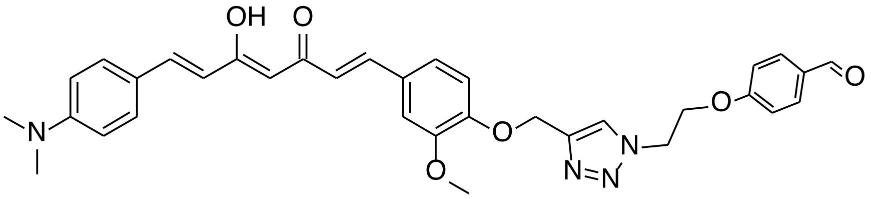

Molecular Formula: $C_{34}H_{34}N_4O_6$
Formula Weight: 594.65696
[M+H]+: 595.255111 Da
[M-H]-: 593.240558 Da

[M+H]⁺ Obt. 595.25479 Calc. 595.25511 ppm 0.61

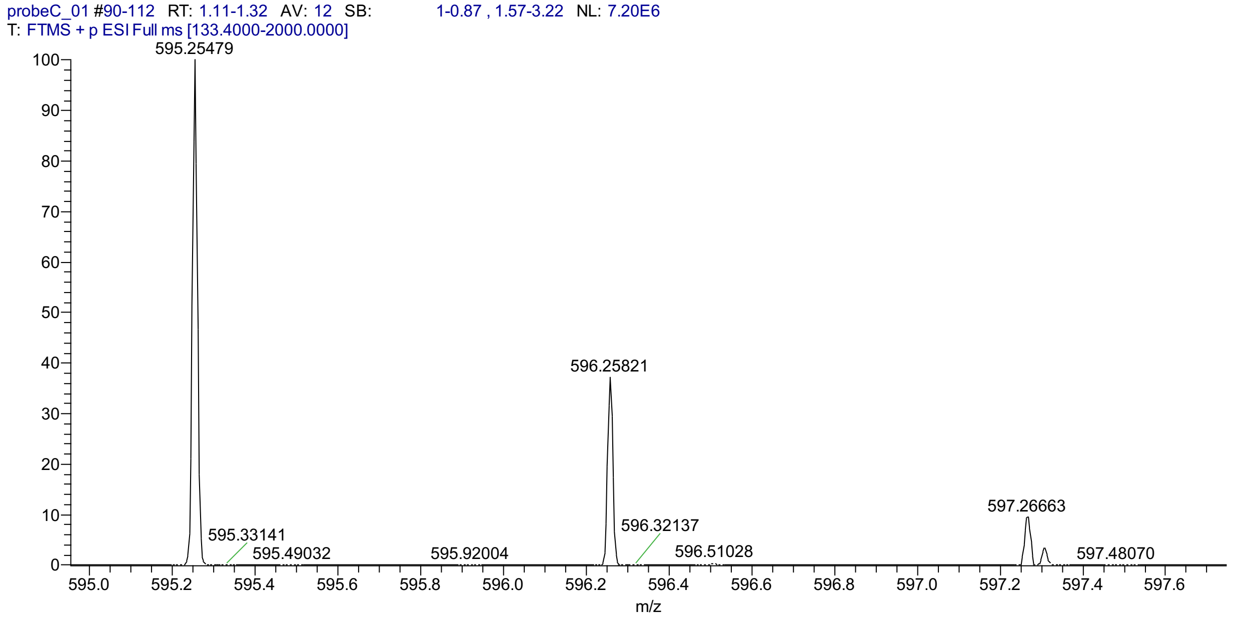

13

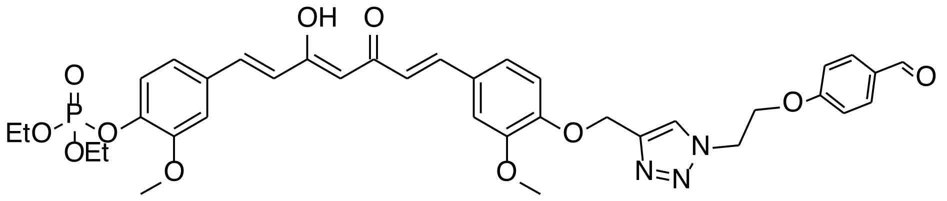

Molecular Formula: $C_{37}H_{40}N_3O_{11}P$
Formula Weight: 733.700762
[M+H]+: 734.247322 Da
[M-H]-: 732.232769 Da

[M+H]$^+$ Obt. 734.24738 Calc. 734.24732 ppm 0.08

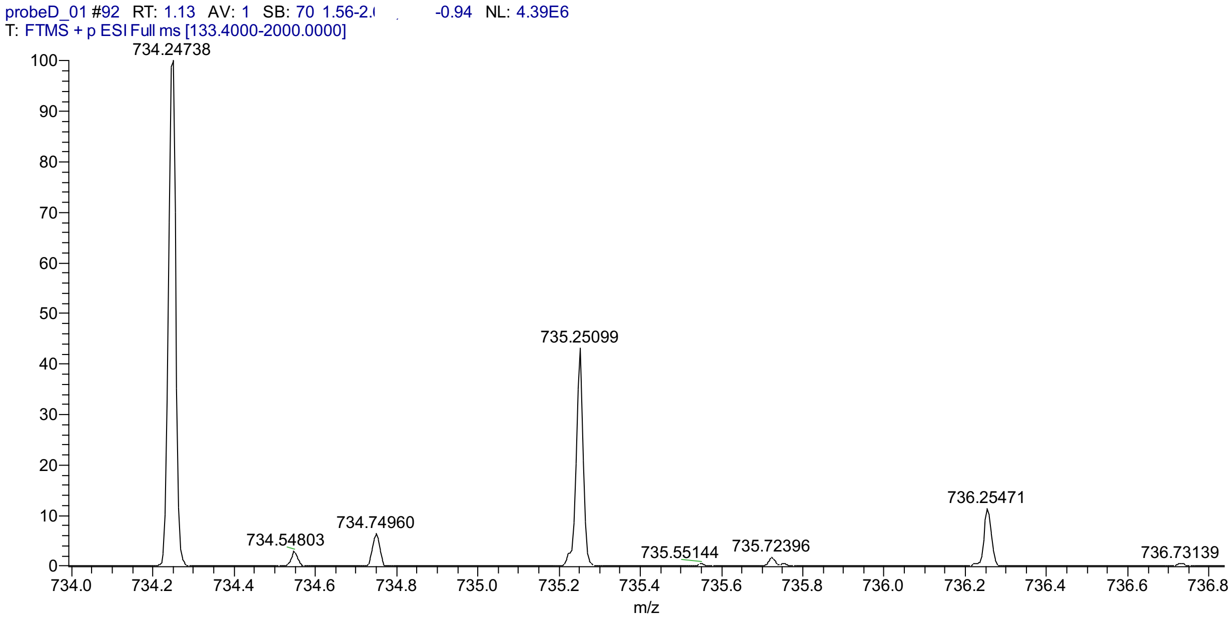

14

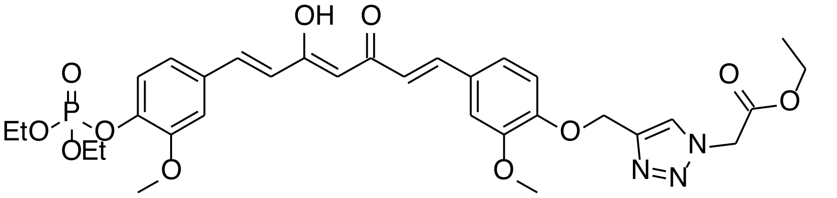

Molecular Formula: $C_{32}H_{38}N_3O_{11}P$
Formula Weight: 671.631382
[M+H]+: 672.231671 Da
[M-H]-: 670.217119 Da

$[M+H]^+$ Obt. 672.23120 Calc. 672.23167 ppm 0,70

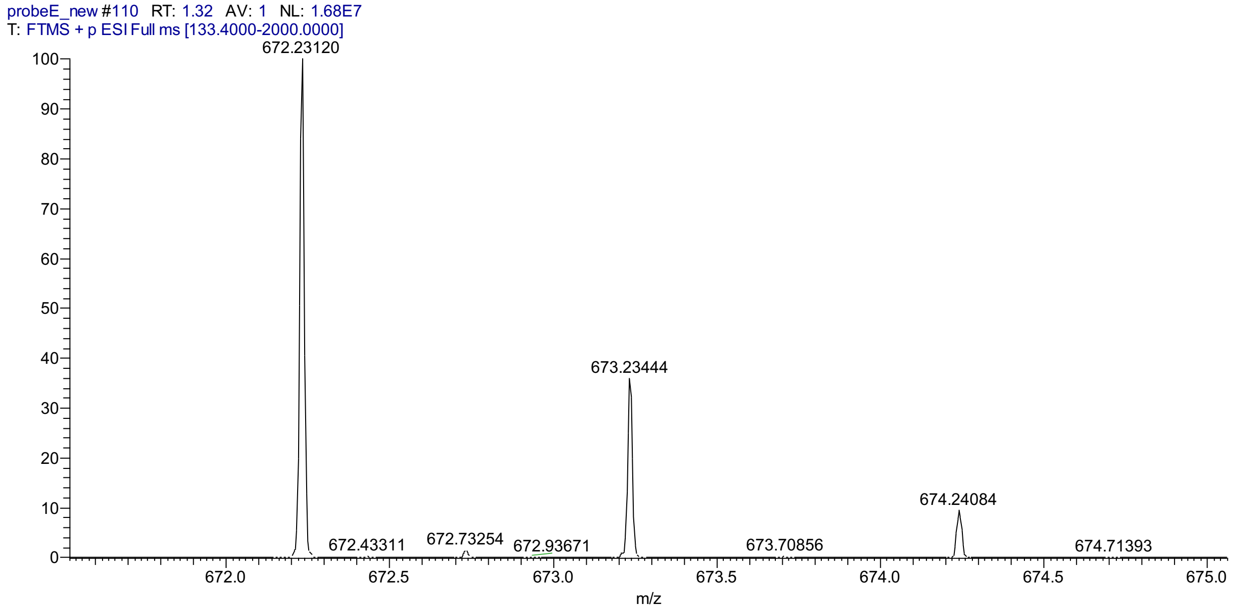

Supplementary Figure S2: LC-HRMS analysis for the determination of interaction of Probe 10 and Probe 11 with the different isoenzymes of ALDH1A

S2.a Chromatograms of the m/z of Probe 10 and Probe 10-COOH in the different samples: A.blank, B. ALDH1A1, C. ALDH1A2 and D. ALDH1A3

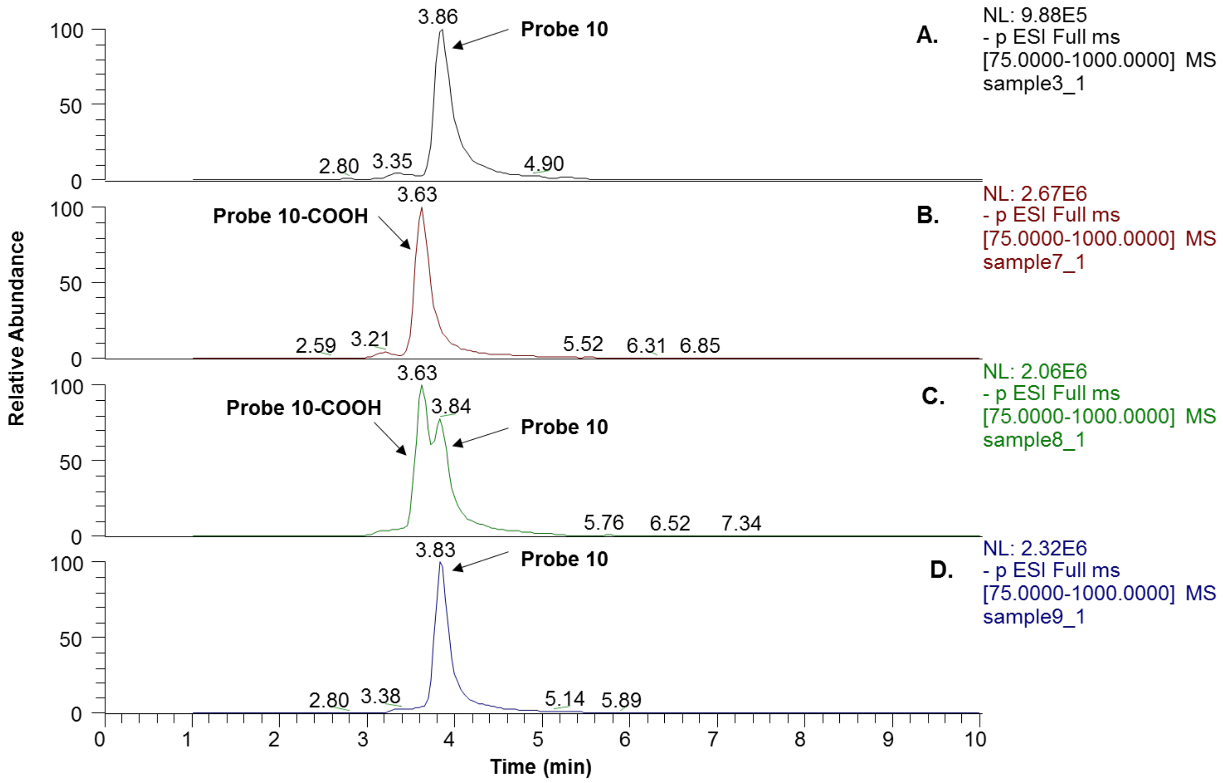

S2.b Chromatograms of the m/z of Probe 11 and Probe 11-COOH in the different samples: A.blank, B. ALDH1A1, C. ALDH1A2 and D. ALDH1A3

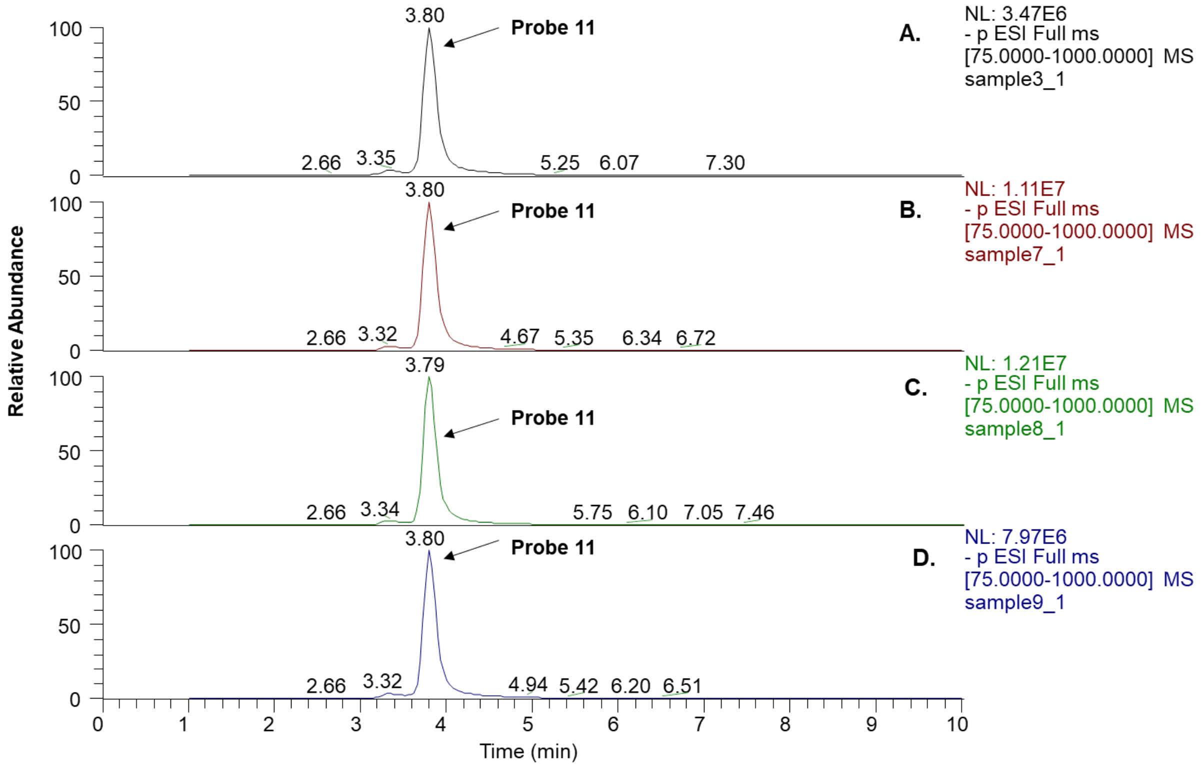

Supplementary Figure S3: Biochemical characterization of the interaction of probe 11 with human ALDH1A3. K_d graph and values with relative R^2 of the complex between Probe **11** at fixed concentration of 10 µM and various concentration of ALDH1A1, ALDH1A2 and ALDH1A3, from 100 µM to 1,1719 µM.

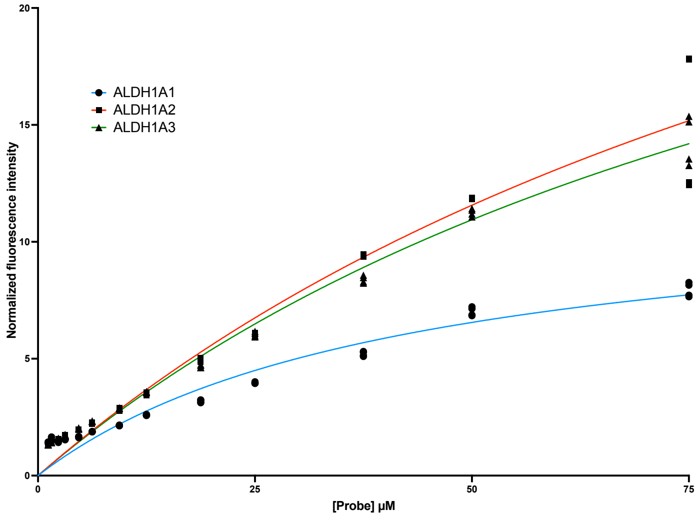

	Kd (µM)	R^2
ALDH1A1	42.4	0.93
ALDH1A2	124.8	0.98
ALDH1A3	84.3	0.97

Supplementary Figure S4: Flow cytometry analysis of Probe 10 on different cell lines. Flow cytometry analysis of Probe **10** on Human 3054, 3060, U87MG ALDH1A3$^+$ glioblastoma cells, HEK293T ALDH1A2$^+$ cell line, human foetal astrocytes (hASTRO) ALDH1A1$^+$ cell line and 4T1 mammary carcinoma as triple negative ALDH1A subfamily.

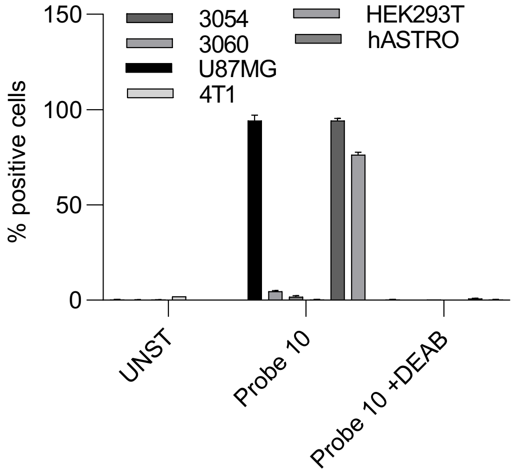

Supplementary Figure S5: Probe 11 is selective on U87 ALDH1A3 positive cells. **a-** Confocal fluorescence microscope images for probe 11 detection in different cell lines at 10 μM, after 2h of incubation. **b-** Cell viability at 10 μM of probe 11. **c-** Relative fluorescence intensity of the images from A. **d-** Flow cytometry analysis of U87-MG cells unstained, stained with probe 11 (10 μM) with or without DEAB (1 μM). (c) Histographic profiles of unstained, stained with probe 11 (10 μM) with or without DEAB (1 μM). P value: **** p<0.0001.

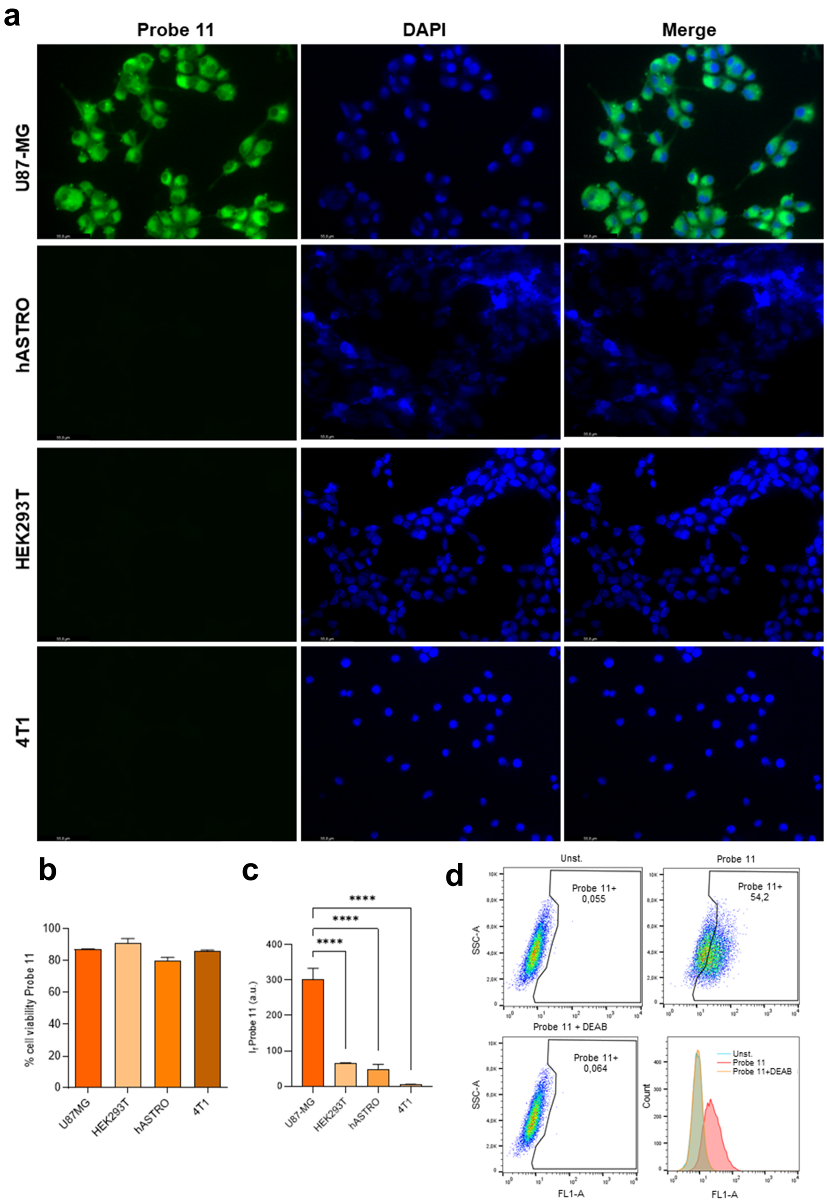

Supplementary Figure S6: *In vitro* experiments showing that ALDH1A3 is the most expressed isoform in GL261 murine glioma cells and Probe 11 enter and label the same cells. Living GL261 cells were analysed through an ImageStreamX MarkII using two channels: brightfield (Ch 01) and fluorescence (Ch 02) after one hour of incubation in Probe **11**, cells were thoroughly washed in PBS and analysed. Two different representative cells are shown. From above, row 1 brightfield images (Ch 01), row 2 fluorescent images (Ch 02). “In Focus Cells” were identified based on the “Gradient Root Mean Square (RMS) Contrast Feature” that captures in focus images of cells identified by high normalized pixel intensity gradient (RMS values) derived from Ch 01; then, a scatter plot of the “Aspect Ratio Feature” versus brightfield “Area Feature” was used to identify single cells (singlets) from debris or cell clumps based on high aspect ratio and low area value. In addition, fluorescence intensity variations are shown in the histogram and dot plot (Area vs Intensity). Scale bars: 5 µm are the same in all images.

probe 11

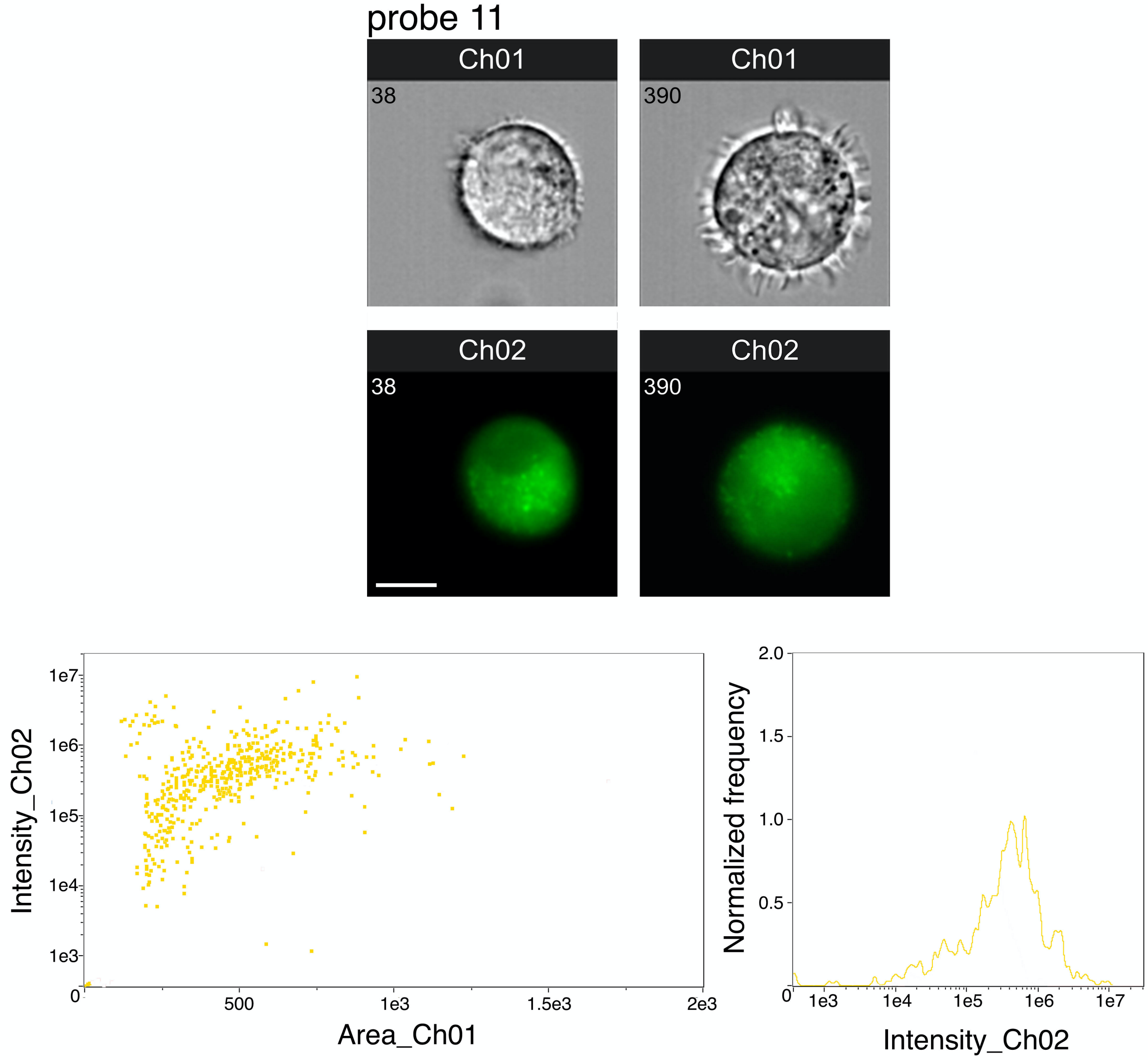

Supplement: Supplementary file 1 — Supplementary Information [file 42003_2022_3834_MOESM1_ESM.pdf]
