# Supplementary material for: Curcumin-based-fluorescent probes targeting ALDH1A3 as a promising tool for glioblastoma precision surgery and early diagnosis
Source: Commun Biol. 2022 Sep 1;5:895. doi: 10.1038/s42003-022-03834-7 (PMC9437101; doi:10.1038/s42003-022-03834-7)
Supplement: Supplementary file 2 — Description of Additional Supplementary Files [file 42003_2022_3834_MOESM2_ESM.pdf]

Description of Additional Supplementary Files

File name: Supplementary Data 1
Description: All raw data of our experiments.
