# Supplementary material for: Curcumin-based-fluorescent probes targeting ALDH1A3 as a promising tool for glioblastoma precision surgery and early diagnosis
Source: Commun Biol. 2022 Sep 1;5:895. doi: 10.1038/s42003-022-03834-7 (PMC9437101; doi:10.1038/s42003-022-03834-7)
Supplement: Supplementary file 4 — Reporting Summary [file 42003_2022_3834_MOESM4_ESM.pdf]

nature research

Corresponding author(s): Silvia Garavaglia
COMMSBIO-21-1351B

Last updated by author(s): Jun 22, 2022

Reporting Summary

Nature Research wishes to improve the reproducibility of the work that we publish. This form provides structure for consistency and transparency in reporting. For further information on Nature Research policies, see our Editorial Policies and the Editorial Policy Checklist.

Statistics

For all statistical analyses, confirm that the following items are present in the figure legend, table legend, main text, or Methods section.

n/a	Confirmed	
☐	☒	The exact sample size (n) for each experimental group/condition, given as a discrete number and unit of measurement
☐	☒	A statement on whether measurements were taken from distinct samples or whether the same sample was measured repeatedly
☐	☒	The statistical test(s) used AND whether they are one- or two-sided<br>*Only common tests should be described solely by name; describe more complex techniques in the Methods section.*
☐	☒	A description of all covariates tested
☐	☒	A description of any assumptions or corrections, such as tests of normality and adjustment for multiple comparisons
☐	☒	A full description of the statistical parameters including central tendency (e.g. means) or other basic estimates (e.g. regression coefficient) AND variation (e.g. standard deviation) or associated estimates of uncertainty (e.g. confidence intervals)
☐	☒	For null hypothesis testing, the test statistic (e.g. F, t, r) with confidence intervals, effect sizes, degrees of freedom and P value noted<br>*Give P values as exact values whenever suitable.*
☒	☐	For Bayesian analysis, information on the choice of priors and Markov chain Monte Carlo settings
☒	☐	For hierarchical and complex designs, identification of the appropriate level for tests and full reporting of outcomes
☒	☐	Estimates of effect sizes (e.g. Cohen's d, Pearson's r), indicating how they were calculated

Our web collection on statistics for biologists contains articles on many of the points above.

Software and code

Policy information about availability of computer code

Data collection	SparkControl™ (Tecan Spark), Magellan (Tecan Sunrise)
Data analysis	FlowJo v10.6.1, GraphPad Prism 9.1., SigmaPlot v9.0

For manuscripts utilizing custom algorithms or software that are central to the research but not yet described in published literature, software must be made available to editors and reviewers. We strongly encourage code deposition in a community repository (e.g. GitHub). See the Nature Research guidelines for submitting code & software for further information.

Data

Policy information about availability of data

All manuscripts must include a data availability statement. This statement should provide the following information, where applicable:

- Accession codes, unique identifiers, or web links for publicly available datasets
- A list of figures that have associated raw data
- A description of any restrictions on data availability

The datasets generated during and/or analysed during the current study are available from the corresponding author on reasonable request

Field-specific reporting

Please select the one below that is the best fit for your research. If you are not sure, read the appropriate sections before making your selection.

☒ Life sciences ☐ Behavioural & social sciences ☐ Ecological, evolutionary & environmental sciences

For a reference copy of the document with all sections, see nature.com/documents/nr-reporting-summary-flat.pdf

Life sciences study design

All studies must disclose on these points even when the disclosure is negative.

Sample size	Sample size was determined empirically for sufficient statistical power. Variations between samples were also used to determine the suitability of the sample size.
Data exclusions	No data were excluded from analysis.
Replication	The number of replicates performed for each experiments were indicated in the figure legends. Data presented are either representative of of replicates experiments of similar finding or average of replicates as indicated.
Randomization	No randomization was performed.
Blinding	The investigator and personnel were not blinded during this study.

Reporting for specific materials, systems and methods

We require information from authors about some types of materials, experimental systems and methods used in many studies. Here, indicate whether each material, system or method listed is relevant to your study. If you are not sure if a list item applies to your research, read the appropriate section before selecting a response.

Materials & experimental systems

n/a	Involved in the study	
☐	☒	Antibodies
☐	☒	Eukaryotic cell lines
☒	☐	Palaeontology and archaeology
☐	☒	Animals and other organisms
☒	☐	Human research participants
☒	☐	Clinical data
☒	☐	Dual use research of concern

Methods

n/a	Involved in the study	
☒	☐	ChIP-seq
☐	☒	Flow cytometry
☒	☐	MRI-based neuroimaging

Antibodies

Antibodies used	Antibodies used in WB have been reported in "Methods". All required information have been provided.
Validation	Antibodies were validated according to the statement reported on the manufacturer's websites.

Eukaryotic cell lines

Policy information about cell lines

Cell line source(s)	U87MG, HEK293T,4T1 cell lines were used and obtained by ATCC. hASTRO was obtained by Prof. Aronica. Patient-derived glioblastoma cell lines 3054 and 3060 obtained by "HGCC" Uppsala University.
Authentication	None of the cell lines used were authenticated in our lab
Mycoplasma contamination	All cell lines used were negative for mycoplasma
Commonly misidentified lines (See ICLAC register)	*Name any commonly misidentified cell lines used in the study and provide a rationale for their use.*

Animals and other organisms

Policy information about studies involving animals; ARRIVE guidelines recommended for reporting animal research

Laboratory animals	Adult 2-3 months old Mus musculus, strain C57BlC
Wild animals	No
Field-collected samples	No
Ethics oversight	All experimental procedures were conducted in accordance with the European Communities Council Directive of the 24th of November 1986 (86/609 EEC), with the Recommendation 18/06/2007, Dir. 2010/63/UE and with the Italian law for care and use of experimental animals (DL116/ 92) and were approved by the Italian Ministry of Health (prot. E669C.15) and by the Bioethical Committee of the University of Turin.

Note that full information on the approval of the study protocol must also be provided in the manuscript.

Flow Cytometry

Plots

Confirm that:

☒ The axis labels state the marker and fluorochrome used (e.g. CD4-FITC).

☒ The axis scales are clearly visible. Include numbers along axes only for bottom left plot of group (a 'group' is an analysis of identical markers).

☒ All plots are contour plots with outliers or pseudocolor plots.

☒ A numerical value for number of cells or percentage (with statistics) is provided.

Methodology

Sample preparation	Cell preparations and staining with antibodies have been described in Methods
Instrument	Cells were acquired using BD LSRFortessa™.
Software	FlowJo v 10.6.1
Cell population abundance	Abundance and purity in the relevant cell populations within post-sort fractions were assessed by flow cytometry analysis. Cells with purity > 90% were used for gene expression analysis.
Gating strategy	The gating strategy is defined in the Figures of the main manuscript

☐ Tick this box to confirm that a figure exemplifying the gating strategy is provided in the Supplementary Information.
